# Mitochondrial fragmentation and network architecture in degenerative diseases

**DOI:** 10.1371/journal.pone.0223014

**Published:** 2019-09-26

**Authors:** Syed I. Shah, Johanna G. Paine, Carlos Perez, Ghanim Ullah

**Affiliations:** Department of Physics, University of South Florida, Tampa, FL, United States of America; Texas Technical University Health Sciences Center, UNITED STATES

## Abstract

Fragmentation of mitochondrial network has been implicated in many neurodegenerative, renal, and metabolic diseases. However, a quantitative measure of the microscopic parameters resulting in the impaired balance between fission and fusion of mitochondria and consequently the fragmented networks in a wide range of pathological conditions does not exist. Here we present a comprehensive analysis of mitochondrial networks in cells with Alzheimer’s disease (AD), Huntington’s disease (HD), amyotrophic lateral sclerosis (ALS), Parkinson’s disease (PD), optic neuropathy (OPA), diabetes/cancer, acute kidney injury, Ca^2+^ overload, and Down Syndrome (DS) pathologies that indicates significant network fragmentation in all these conditions. Furthermore, we found key differences in the way the microscopic rates of fission and fusion are affected in different conditions. The observed fragmentation in cells with AD, HD, DS, kidney injury, Ca^2+^ overload, and diabetes/cancer pathologies results from the imbalance between the fission and fusion through lateral interactions, whereas that in OPA, PD, and ALS results from impaired balance between fission and fusion arising from longitudinal interactions of mitochondria. Such microscopic difference leads to major disparities in the fine structure and topology of the network that could have significant implications for the way fragmentation affects various cell functions in different diseases.

## Introduction

Mitochondrion is a ubiquitous organelle and powerhouse of the cell that exists in living cells as a large tubular assembly, extending throughout the cytoplasm and in close apposition with other key organelles such as nucleus, the endoplasmic reticulum, the Golgi network, and the cytoskeleton [1–5]. Its highly flexible and dynamic network architecture ranging from a few hundred nanometers to tens of micrometers with the ability to rapidly change from fully connected to fragmented structures makes it suitable for diverse cytosolic conditions and cell functions [6–8]. Cells continuously adjust the rate of mitochondrial fission and fusion in response to changing energy and metabolic demands to facilitate the shapes and distribution of mitochondria throughout the cell [9–11]. Similarly, stressors such as reactive oxygen species (ROS) and Ca^2+^ dysregulation interfere with various aspects of mitochondrial dynamics [12–14]. This is probably why many neuronal, metabolic, and renal diseases have been linked to primary or secondary changes in mitochondrial dynamics [9, 15–37]. Neuronal cells, due to their complex morphology and extreme energy dependent activities such as synaptic transmission, vesicle recycling, axonal transport, and ion channels and pumps activity, are particularly sensitive to changes in the topology of mitochondrial network [38–41].

The mitochondrial network organization makes a bidirectional relationship with the cell’s bioenergetics and metabolic variables [11, 42]. For example, the morphological state of mitochondria has been linked to their energy production capacity [43–46], as well as cell health and death [10, 46–49] on one hand, alterations in mitochondrial energy production caused by genetic defects in respiratory chain complexes lead to fragmentation of mitochondrial network [50, 51] on the other hand. Similarly, while ROS induces fragmentation of mitochondrial network [12–14], overproduction of ROS in hyperglycemic conditions requires dynamic changes in mitochondrial morphology and fragmentation of the network [52]. Furthermore, high cytosolic Ca^2+^ induces mitochondrial fragmentation [14], whereas fragmentation blocks the propagation of toxic intracellular Ca^2+^ signals [53, 54] and can limit the local Ca^2+^ uptake capacity of mitochondria due to their smaller sizes. Thus dynamic changes in mitochondrial morphology and fragmentation of its network can be part of the cycle that drives the progression of degenerative diseases [11–13, 18, 22, 52, 55–70].

Despite a clear association with many cell functions in physiological conditions, quantitative measures of the microscopic fission and fusion rates leading to a given topology of the mitochondrial network remain elusive. While fluorescence imagining has been instrumental in providing biologically useful insights into the structure and function of mitochondria, detailed description of the kinetics and the dynamical evolution of the complex mitochondrial networks in health and disease are still out of reach of these techniques. Although it is difficult to study such dynamics experimentally, computational techniques provide a viable alternative. Various computational studies on the identification and analysis of network parameters from experimental mitochondrial micrographs have been performed using either custom built applications [71–76] or commercially available tools [77], depending upon the particular question being asked. However, a comprehensive study quantifying the imbalance between fission and fusion responsible for the network fragmentation observed in many diseases does not exist.

In this paper, we adopt and extend the method developed in Refs. [75, 76] using a pipeline of computational tools that process and extract a range of network parameters from mitochondrial micrographs recorded through fluorescence microscopy, and simulate mitochondrial networks to determine microscopic rates of fission and fusion leading to the observed network properties. We first demonstrate our approach by application to images of mitochondrial networks in striatal cells from YAC128 Huntington’s disease (HD) transgenic mice (bearing a 111 polyglutamine repeat Q111/0 and Q111/1) and their control counterparts reported in Ref. [78]. This is followed by the application of our technique to images of mitochondria in cells with Alzheimer’s disease (AD) [79], amyotrophic lateral sclerosis (ALS) [80], Parkinson’s disease (PD) [81], optic neuropathy (OPA) [66], diabetes/cancer [65], acute kidney injury [64], Ca^2+^ overload [14], and Down syndrome (DS) [36, 82] pathologies from the literature. The images analyzed in this study were selected based on the following criteria. (1) The paper from which the images were selected reported images of mitochondrial networks both in normal and diseased cells from the same cell/animal model. (2) The images were of high enough quality so that they can be processed properly, making sure that the network extracted indeed represented the actual mitochondrial network without introducing artifacts during the processing. The cell/animal models used in these studies are listed in S1 Table in the Supplementary Information Text and detailed in the Results section below. Although we found fragmented mitochondrial networks and imbalanced fission and fusion in all these pathologies in comparison to their respective control conditions, significant differences between the microscopic properties underlying such fragmentation exist in different diseases.

## Methods

### Image analysis

Mitochondria in a cell can form networks of different topologies ranging from a fully disintegrated network with one mitochondrion per cluster to a well-connected network comprising of clusters with several mitochondria per cluster to a fully connected network where all clusters are connected to form a single giant cluster. These topologies can be uniquely distinguished by various network parameters such as the mean degree <k> (the average number of nearest neighbors), giant cluster N_g_ (the largest cluster in the network), giant cluster normalized with respect to the total number of nodes (mitochondria) or edges (connections) N_g_/N, and distributions of various features such as the number of mitochondria in various linear branches, cyclic loops, and clusters comprising both branches and loops.

**Fig 1:**
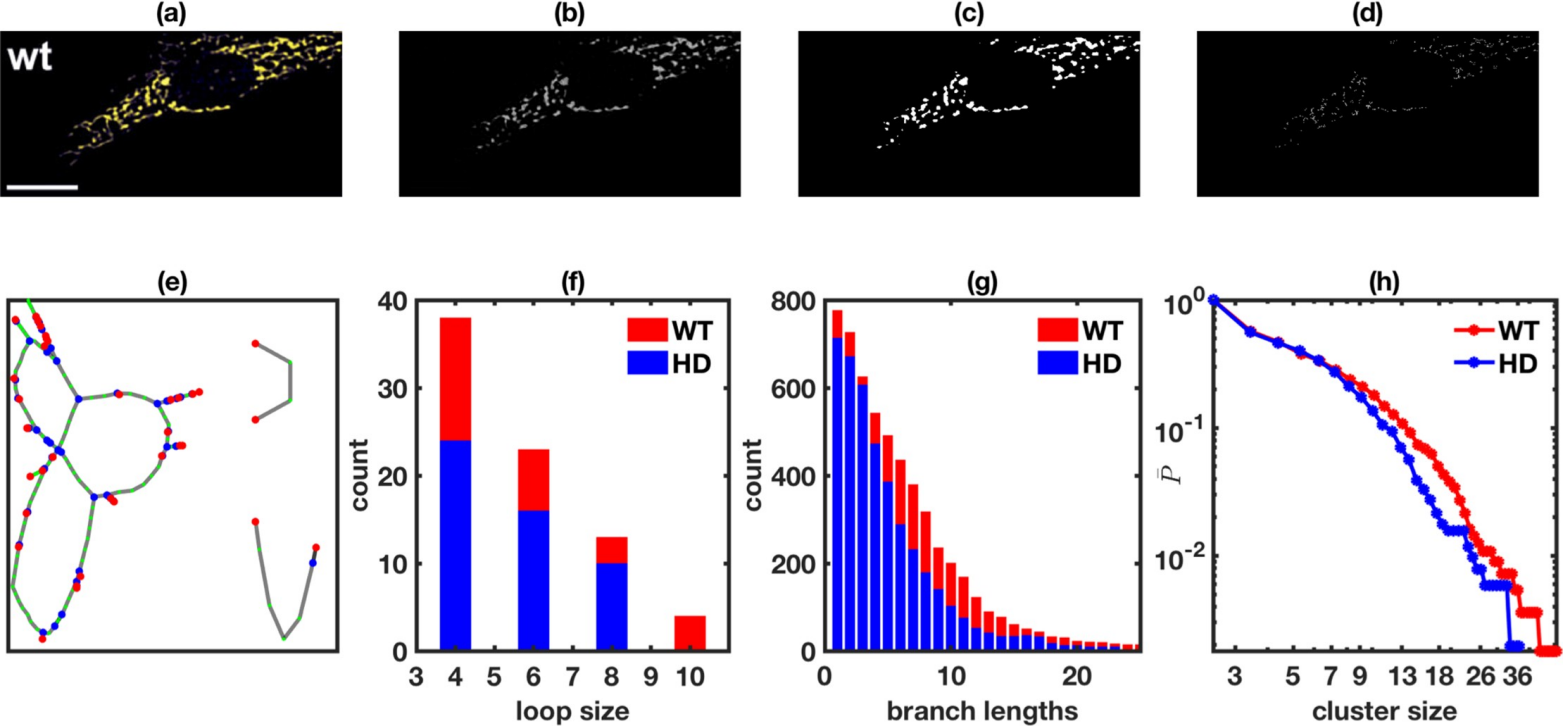
Steps involved in the processing of the images and retrieval of various network features. (a) Original image, (b) the grayscale image containing mitochondrial network only, (c) binary image, and (d) skeletonized image. Panel (e) shows a graph (partially shown) representation of the skeletonized image where red, green, and blue colors represent nodes with degree 1, 2 and 3 respectively. Size distribution of cyclic loops (f) and linear branch lengths (g), and cumulative probability distribution of cluster sizes (h) in mitochondrial network in striatal cells from wildtype (NL, red) and YAC128 HD (blue) transgenic mice. The image used for the mitochondrial network extraction in panel (a) was adopted from Ref. [78] with permission.

To extract all this information from experimental images of mitochondrial networks, we adopt and extend the procedure first reported in Ref. [75] using a pipeline of Matlab (The MathWorks, Natick, MA) tools. Often, we are required to preprocess the images for removing any legends or masking/removing areas that contain artifacts (Fig 1A). The colors representing processes other than mitochondria are removed and the resulting image is converted to grayscale image (Fig 1B). Next, we take a series of steps to extract the underlying mitochondrial network and the key information about the network.

Step 1: We use Matlab function *im2bw* to generate a binary image (Fig 1C) from the preprocessed gray scale image (Fig 1B) of the micrograph by applying appropriate threshold intensity using Matlab function *graythresh*.

Step 2: The resulting binary image is reduced to a trace of one-pixel thick lines called skeleton using Matlab function *bwmorph*, which represents mitochondrial network (Fig 1D).

Step 3: To extract various features of the mitochondrial network from skeletonized image, we first label different clusters using Matlab routine *bwlabel*. The labeled clusters are then converted to a graph (Fig 1R, only partial graph is shown for clarity) where the nodes are color-coded according to their degree. The graph is then used to extract network parameters such as <k>, N_g_, and N_g_/N. We also extracted size distribution of loops or cycles with no open ends (Fig 1F), size distribution of branches with at least one open end (Fig 1G), and cumulative probability distribution of individual cluster sizes (Fig 1H) in terms of number of edges, where a single cluster could have both loops and branches and is disconnected from other clusters.

All the above properties are extracted for mitochondrial networks in the cells with different pathologies and the corresponding control cells for comparison. For example, we compare the size distributions of loops, branches, and clusters in striatal cells from YAC128 Huntington’s disease (HD) transgenic mice (blue) and their control counterparts (NL, red) reported in Ref. [78] in Fig 1F–1H. A clear leftward shift in these distributions can be seen in HD, indicating a fragmented mitochondrial network as compared to NL cells. The overall number of loops and branches also decreases in HD.

### Modeling and simulating mitochondrial network

**Fig 2:**
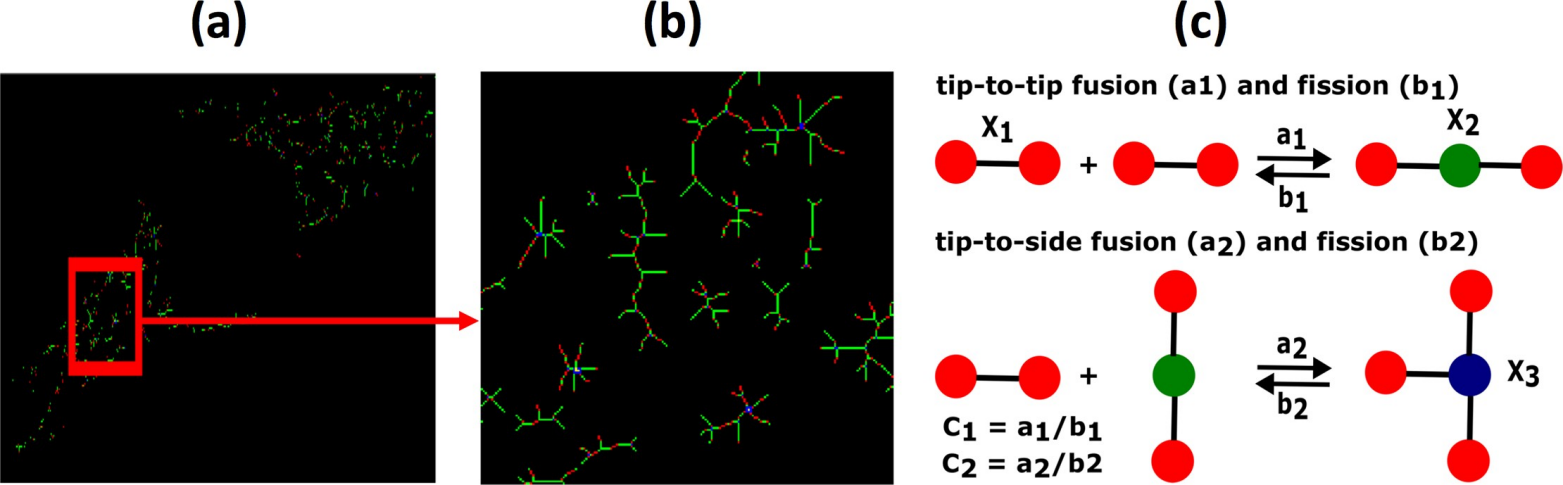
Experimentally observed mitochondrial network and the scheme to model it. (a) Color coded mitochondrial network retrieved from experimental image of a striatal cell from a wildtype mice and (b) its zoomed in version. (c) Model scheme representing the tip-to-tip fusion of two X1 nodes into X2 and tip-to-side fusion of one X1 node with one X2 node to make one X3 node, and their corresponding fission processes. The image used for the mitochondrial network extraction in panel (a) was adopted from Ref. [78] with permission.

To simulate mitochondrial network, we used the model described in Sukhorukov *et al*. [76], where the network results from two fusion and two fission reactions (Fig 2). In the model, a dimer tip representing a single mitochondrion can fuse with other dimer tips, forming a network node. At most three tips can merge. The two possible fusion and corresponding fission reactions are termed as tip-to-tip and tip-to-side reactions. The biological equivalent of the tip-to-tip reaction would be the fusion of two mitochondria moving along the same microtubule track in the opposite directions and interacting longitudinally [83]. Similarly, tip-to-side reaction mimics the merging of two mitochondria moving laterally [83]. These two types of interactions are explained further in section “Mitochondrial interactions” of Supplementary Information text and sketched in S1 Fig. This way, the network can have nodes with degree 1 (isolated tip), degree 2 (two merged nodes), and degree 3 (three merged nodes). To each fusion process, there is an associated fission process. Thus, the four possible processes can be represented by the following two reaction equations.

$$X1+X1\begin{array}{c} \overset{a1}{\to }\\ \underset{b1}{\leftarrow } \end{array}X2,$$

$$X1+X2\begin{array}{c} \overset{a2}{\to }\\ \underset{b2}{\leftarrow } \end{array}X3.$$

Where X1 (Fig 2A, red), X2 (Fig 2A, green), and X3 (Fig 2A, blue) represent nodes with degree 1, 2, and 3 respectively. Nodes with degree 4 are not included because of their extremely low probability [75, 76]. Network edges connecting the nodes define minimal (indivisible) constituents of the organelle. Therefore, all parameters are calculated in terms of number of edges in the network.

Next, we implement the model as an agent-based model using Gillespie algorithm [75, 76, 84]. We initialize the simulation with the number of edges (N) estimated from experimental micrographs of the cell that we intend to model and all nodes initially in X1 form with their number equal to the mitochondrial components representing the cell. The number of edges in the images processed in this paper ranges from as few as 72 to as many as 19519. The network is allowed to evolve through a sequence of fusion and fission processes according to their propensities at a given time step. In all cases, we run the algorithm for 5N time steps to reach the steady state and extract various network features (<k>, N_g_, branch lengths etc.) at the end of the run using various Graph and Network algorithms in Matlab. Depending on the fusion (a1 & a2) and fission (b1 & b2) rates used, networks of varying properties ranging from mostly consisting of isolated mitochondria or branched clusters to a fully connected one giant cluster can be generated [76].

**Fig 3:**
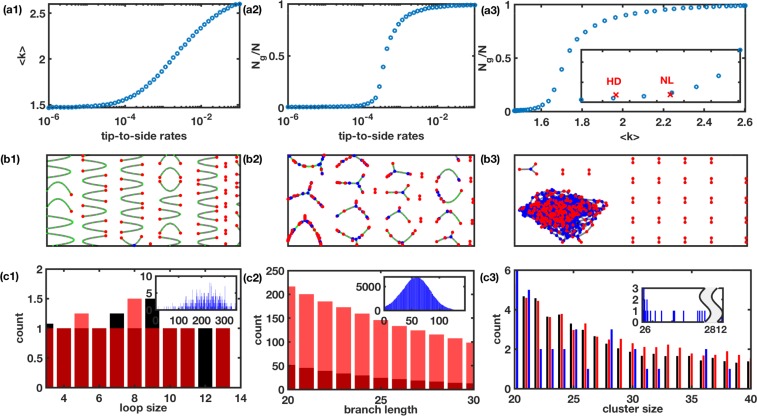
Model results at different C1 and C2 values. Mean degree (a1), N_g_/N (a2), and N_g_/N versus <k> (a3) as functions of C2 at a fixed value of C1. Inset in (a3) shows a zoomed in version of the main plot in (a3) with superimposed N_g_/N versus <k> from experimental images of mitochondria in striatal cells (red cross) from wildtype (NL) and YAC128 HD transgenic mice [78]. Mitochondrial network changes from fragmented (b1) to physiologically viable, well-connected (b2) to a fully connected network making one giant cluster (b3) as we increase C2 (or C1). Distribution of loop sizes (c1), branch lengths (c2), and cluster sizes (c3) retrieved from simulated networks at two different C2 values corresponding to mitochondrial network in striatal cells from HD transgenic mice (representative of low C2) (black bars) and striatal cells from wildtype mice in the same experiments (representative of intermediate C2) (red bars). The insets in (c1) and (c2) and the blue bars in (c3) correspond to C2 value for the normal cells in ALS experiments (representative of high C2). The inset in (c3) shows the tail of the blue distribution indicating the formation of a giant cluster at high C2. At smaller cluster sizes, the black, red, and blue bars in panel (c1) are comparable and are skipped for clarity.

To search for a network with specific properties, we follow the procedure in [75, 76] and vary the ratio of fusion and fission processes, i.e. C1 = a1/b1 and C2 = a2/b2 by fixing b1 and b2 at 0.01 and 3b1/2 respectively, and allowing a1 and a2 to vary. For every set of (C_1_, C_2_) values, we repeat the simulations 100 times with different sequences of random numbers and report different parameters/features of the network averaged over all 100 runs. Results from a sample run with N = 3000 are shown in Fig 3A1–3A3, where we plot <k> (Fig 3A1) and N_g_/N (Fig 3A2) as functions of C2 at fixed C1 = 0.0007. N_g_/N versus <k> from the same simulation is shown in Fig 3A3. Increasing C1 shifts the curve to the right. We scan a wide range of C1 and C2 values and plot <k> and N_g_/N obtained from experimental images on this two parameter phase space diagram. As an example, the red crosses in the inset in Fig 3A3 represent N_g_/N versus <k> retrieved from experimental images of mitochondria in striatal cells from NL and HD transgenic mice [78]. The values from the image are mapped with the corresponding C1 and C2 values on the phase space diagram and reported as the values for that cell.

Larger values of C1 and C2 mean more frequent tip-to-tip and tip-to-side fusion respectively, and vice versa. A very small value of C2 (or C1) results in a network mainly consisting of linear chains and isolated nodes (Fig 3B1) with small <k> and N_g_/N (Fig 3A1 & 3A2). Medium value of C2 leads to a network having clusters with both branches and loops (Fig 3B2), whereas large C2 value results in a network having one giant cluster (Fig 3B3) with large <k> and N_g_/N values. To demonstrate further that how low, intermediate, and large values of C2 (or C1) affect the fine structure of the network, we show distributions of the loop, branch, and cluster sizes from three simulations in Fig 3C1–3C3. We pick C2 values obtained for mitochondrial networks (details about C1 and C2 values for different conditions are given below) in striatal cells with HD pathology (C2 = 0.22e-4, C1 = 4.9e-4), their corresponding NL cells (C2 = 0.44e-4, C1 = 4.9e-4) [78], and NL cells from ALS experiments (C2 = 1.0e-4, C1 = 4.8e-4) reported in Ref. [80] as representatives of the three cases. We also performed simulations using C1 and C2 values representing mitochondrial networks in cells with DS pathology (C2 = 0.32e-4 value) and their corresponding NL cells (C2 = 0.88e-4 value) [36, 82] and observed a clear rightward shift in all three distributions at 0.88e-4 as compared to those at C2 = 0.32e-4 (not shown). In addition to shifting to the right, the range of all three distributions widens as we increase the value of C2, indicating that both the sizes and diversity of the network components increase.

## Results

As pointed out above, we processed images of mitochondrial networks in cells with various neurological pathologies including AD [79], ALS [80], PD [81], HD [78], OPA [66], Ca^2+^ overload in astrocytes [14], and DS [36, 82] as well as other conditions such as kidney disease [64] and diabetes/cancer [65] from published literature. Details of the cell models analyzed are given in the following paragraphs and tabulated in S1 Table. Key network parameters such as <k>, N_g_, N_g_/N retrieved from the diseased cells and their normal counterparts are listed in Table 1. A universal signature of all pathological conditions we analyzed in this study is that mitochondrial networks in the diseased cells are fragmented as compared to normal cells. In terms of network parameters, this translates into smaller <k>, average cluster size, N_g_, and N_g_/N for mitochondrial networks in cells with pathological conditions as compared to control cells.

**Table 1:** Network parameters obtained from images of cells with different pathologies. Column 1 lists the disease for which micrographs of normal (NL) and diseased cells were analyzed (column 2). Column 3–8 lists the total number of edges, mean degree, total number of clusters (excluding isolated nodes), average cluster size (in terms of number of edges), giant cluster size (in terms of number of edges), and the ratio of the giant cluster and network size.

Condition	Normal vs diseased	Number of edges	Mean degree <k>	Number of clusters	Avg. cluster size	N_g_	N_g_/N
HD	NL	2664	1.67	556	4.79	50	0.0188
	HD	2150	1.63	512	4.20	40	0.0186
AD	NL	642	1.64	144	4.46	27	0.042
	AD	1061	1.62	258	4.11	40	0.038
DS	NL	1916	1.52	623	3.08	34	0.017
	DS	1365	1.47	502	2.72	14	0.010
PD	NL	19519	1.72	3416	5.71	107	0.006
	PD	8715	1.70	1691	5.15	45	0.005
ALS	NL	103	1.75	19	5.42	38	0.369
	ALS	72	1.69	13	5.54	16	0.222
Kidney injury	NL	5038	1.66	1061	4.75	58	0.012
	Kidney injury	5386	1.64	1207	4.64	59	0.011
Diabetes/Cancer	NL	3546	1.67	715	4.96	82	0.023
	Diabetes/Cancer	3504	1.65	769	4.55	35	0.010
OPA	NL	5263	1.69	1045	5.04	49	0.0093
	OP	7772	1.67	1656	4.69	43	0.0055
Ca^2+^	NL	3195	1.59	903	3.54	126	0.039
	Ca^2+^ overload	2576	1.57	764	3.37	82	0.032

Our observations are in agreement with previous studies investigating these diseases individually. For example, it has been shown that mitochondrial dysfunction in fibroblasts from human fetuses with trisomy of Hsa21 (DS-HFF) [82], human fibroblasts from subjects with DS [36], and mouse embryonic fibroblasts derived from a DS mouse model [36] are correlated with the significant fragmentation of the underlying mitochondrial network when compared to healthy cells, in line with our results showing that <k> and N_g_/N for the network in NL cells are higher than those in DS affected cells. Another study investigating mitochondrial dynamics in AD showed that neuroblastoma cells overexpressing APPswe mutant and amyloid β display more fragmented mitochondrial networks as compared to control cells [79]. Along similar lines, cells with HD pathology were shown to be accompanied by mitochondrial fragmentation and cristae alterations in several cellular models of the disease. These alterations were attributed to increased basal activity of the Ca^2+^-dependent phosphatase calcineurin that dephosphorylates the pro‐fission dynamin related protein 1 (Drp1) and mediates its translocation to mitochondria [85]. This study also showed that the upregulation of calcineurin activity results from the higher Ca^2+^ concentration in the cytoplasm in HD due to enhanced release from intracellular stores such as the endoplasmic reticulum. Parkinson’s disease is another complex multifactorial etiology, involving many genetic and environmental factors over the course of time. An in-depth analysis of the human primary skin fibroblasts obtained from sporadic late-onset PD patients with those from healthy age-matched control subjects showed that the diseased fibroblasts exhibit significantly compromised mitochondrial structure and function [81]–in line with the network parameters estimated in our study.

We also analyzed images of mitochondrial networks in mouse hippocampus-derived neuroblastoma cells, transduced with wildtype, R15L, and S59L mutations of Coiled-coil-helix-coiled-coil-helix domain-containing protein 10 (CHCHD10) that were reported in Ref. [80]. Both <k> and N_g_ (and N_g_/N) decrease in the presence of CHCHD10 mutations as compared to wildtype CHCHD10. CHCHD10 mutations are associated with a spectrum of familial and sporadic frontotemporal dementia-ALS diseases [86, 87], Charcot–Marie–Tooth disease type 2 [88], mitochondrial myopathy and spinal muscular atrophy Jokela type [89]. Recently, Woo *et al*. [80] showed that CHCHD10 results in cytoplasmic accumulation of TAR DNA-binding protein 43 (TDP-43) that increases mitochondrial fission proteins Drp1 and Fis1, reduces mitochondrial fusion protein Mfn1, and promotes mitochondrial fragmentation [90, 91]. TDP-43 pathology is associated with the vast majority of ALS and frontotemporal lobar degenerations [92] and plays a major role in other neurodegenerative diseases [93, 94] and cellular toxicity in general [95, 96]. Overexpression of TDP-43 also promotes juxtanuclear aggregation of mitochondria [90, 91]. The larger average cluster size we observe in cells with CHCHD10 mutations as compared to NL cells could reflect this behavior (Table 1, column 6).

Mitochondrial damage is also believed to be a key contributor to renal diseases like acute kidney injury. By processing images of mitochondrial networks reported in Brooks *et al*. [64], we observe smaller <k> and N_g_/N in rat proximal tubular cells and primary renal proximal tubular cells treated with azide to induced ATP depletion and model *in vivo* ischemia. These values confirm the conclusions in Ref. [64], where a larger number of cells exhibited fragmented mitochondrial networks in cells treated with azide and cisplatin to induce nephrotoxicity as compared to control cells. The same study also reported that both ischemic acute kidney injury and tubular apoptosis were observed to be ameliorated by Mdivi-1, a pharmacological inhibitor of Drp1.

A dimeric mitochondrial outer membrane protein, MitoNEET, is implicated in the etiology of many pathologies including obesity, insulin resistance, diabetes, and cancer. Its downregulation reduces cell proliferation and tumor growth in breast cancer adipocytes and in pancreatic cells [97–100]. Our analysis of fluorescence images of MitoNEET knockout mouse embryonic fibroblasts indicates that <k>, average cluster size, and N_g_/N all decrease when compared with control mouse embryonic fibroblasts. These results are in agreement with the observations suggesting that the downregulation of MitoNEET in mouse embryonic fibroblasts and pancreatic β cells results in reduced connectivity of mitochondrial network and vice versa [99, 101].

Mitochondriopathies are also associated with many multisystemic diseases including infantile-onset developmental delay, muscle weakness, ataxia, and optic nerve atrophy caused by a homozygous mutation in the yeast mitochondrial escape 1-like 1 gene (YME1L1) [102]. YME1L1 plays a key role in mitochondrial morphology by mediating optic atrophy type 1 (OPA1) protein that is involved in mitochondrial fusion and remodeling, and is also believed to be associated with hereditary Spastic Paraplegia 7 disease, Autosomal Recessive disorder, obesity, and defective thermogenesis [73, 103–106]. We found that <k>, mean cluster size, and N_g_/N all decrease in cells expressing YME1L1 missense mutation R149W and YME1L1. These results are in agreement with the observations of fragmented mitochondrial network in HeLa cells and fibroblasts from mouse and patients with proliferation defect expressing R149W or YME1L1 knockout cells [66] and SHSY5Y cells where YME1L1 is degraded in response to distinct cellular stresses that depolarize mitochondria and deplete cellular ATP [103].

Interestingly, a common feature among the pathological conditions discussed in this paper and several other degenerative diseases where mitochondrial fragmentation is observed, is that intracellular Ca^2+^ concentration in the cells affected by these pathologies is upregulated [107–120]. Therefore, we analyzed images of mitochondrial networks in cells with higher intracellular Ca^2+^ concentration. These images were reported in Ref. [14], where rat cortical astrocytes were treated with Ca^2+^ ionophore 4Br-A23187 that increases intracellular Ca^2+^ concentration in dose-dependent manner. We found that <k>, average cluster size, N_g_, and N_g_/N for mitochondrial network in astrocytes exposed to 4Br-A23187 are significantly lower than those observed in control cells.

Next, we perform extensive stochastic simulations (see “Modeling and simulating mitochondrial network” section) to search for the tip-to-tip and tip-to-side fusion and fission rates characterizing mitochondrial networks in cells with different pathologies and their respective control conditions. Final results from these simulations are summarized in Table 2. As is evident from columns 7 and 8, in all cases the values of C1 or/and C2 for mitochondrial network in diseased cells are smaller than those in control cells. This confirms the lower tip-to-tip or tip-to-side fusion to fission ratios in the diseased cells.

**Table 2:** Comparison of microscopic parameters of mitochondrial network obtained from simulations and experiments. Column 1 lists the condition for which images of normal (NL) and diseased cells were analyzed (column 2). Columns 3 & 4 and 5 & 6 compare <k> and N_g_/N respectively from experiment and theory. Columns 7 & 8 are the C1 (tip-to-tip fusion/fission) and C2 (tip-to-side fusion/fission) values obtained by fitting the model to the data and used in simulations.

Condition	Normal vs diseased	Mean degree <k> Exp Theory		N_g_/N Exp Theory		C_1_	C_2_
HD	NL	1.67	1.67	0.0188	0.022	4.9e-4	4.40e-5
	HD	1.63	1.63	0.0186	0.0101	4.9e-4	2.20e-5
AD	NL	1.64	1.64	0.042	0.108	7.0e-4	2.30e-4
	AD	1.62	1.62	0.038	0.067	7.0e-4	1.90e-4
DS	NL	1.52	1.52	0.017	0.017	5.0e-4	0.88e-4
	DS	1.47	1.47	0.010	0.010	5.0e-4	0.32e-4
PD	NL	1.72	1.72	0.006	0.008	1.2e-3	7.00e-6
	PD	1.70	1.70	0.005	0.007	9.8e-4	7.00e-6
ALS	NL	1.75	1.75	0.369	0.359	4.8e-4	1.00e-4
	ALS	1.69	1.69	0.222	0.225	1.0e-4	1.00e-4
Kidney injury	NL	1.66	1.66	0.012	0.016	9.1e-4	4.00e-5
	Kidney injury	1.64	1.64	0.011	0.011	9.0e-4	0.25e-4
Diabetes/Cancer	NL	1.67	1.67	0.023	0.020	9.8e-4	4.50e-5
	Diabetes/Cancer	1.65	1.65	0.010	0.013	9.8e-4	2.50e-5
OPA	NL	1.69	1.69	0.0093	0.0081	9.0e-4	1.00e-5
	OPA	1.67	1.67	0.0055	0.0075	7.6e-4	1.00e-5
Ca^2+^	NL	1.59	1.59	0.039	0.038	7.0e-4	1.46e-4
	Ca^2+^ overload	1.57	1.58	0.032	0.032	7.0e-4	1.13e-4

In most cases, we identified C1 and C2 where the model gives the exact <k> and N_g_/N values observed in the experiment. In some cases, the N_g_/N value from simulation is slightly different than that retrieved from experimental images. However, it is possible to get C1 and C2 values that would result in the exact N_g_/N values. This will require running the algorithm with smaller C1 and C2 increments, which will significantly increase computational time. On average, simulating the network with one set of C1 and C2 values and 100 repetitions to minimize the stochastic variability, takes 5 to 10 hours (depending on N). Thus, halving the increments of one or both of C1 and C2 would double or quadruple the computational time respectively.

**Fig 4:**
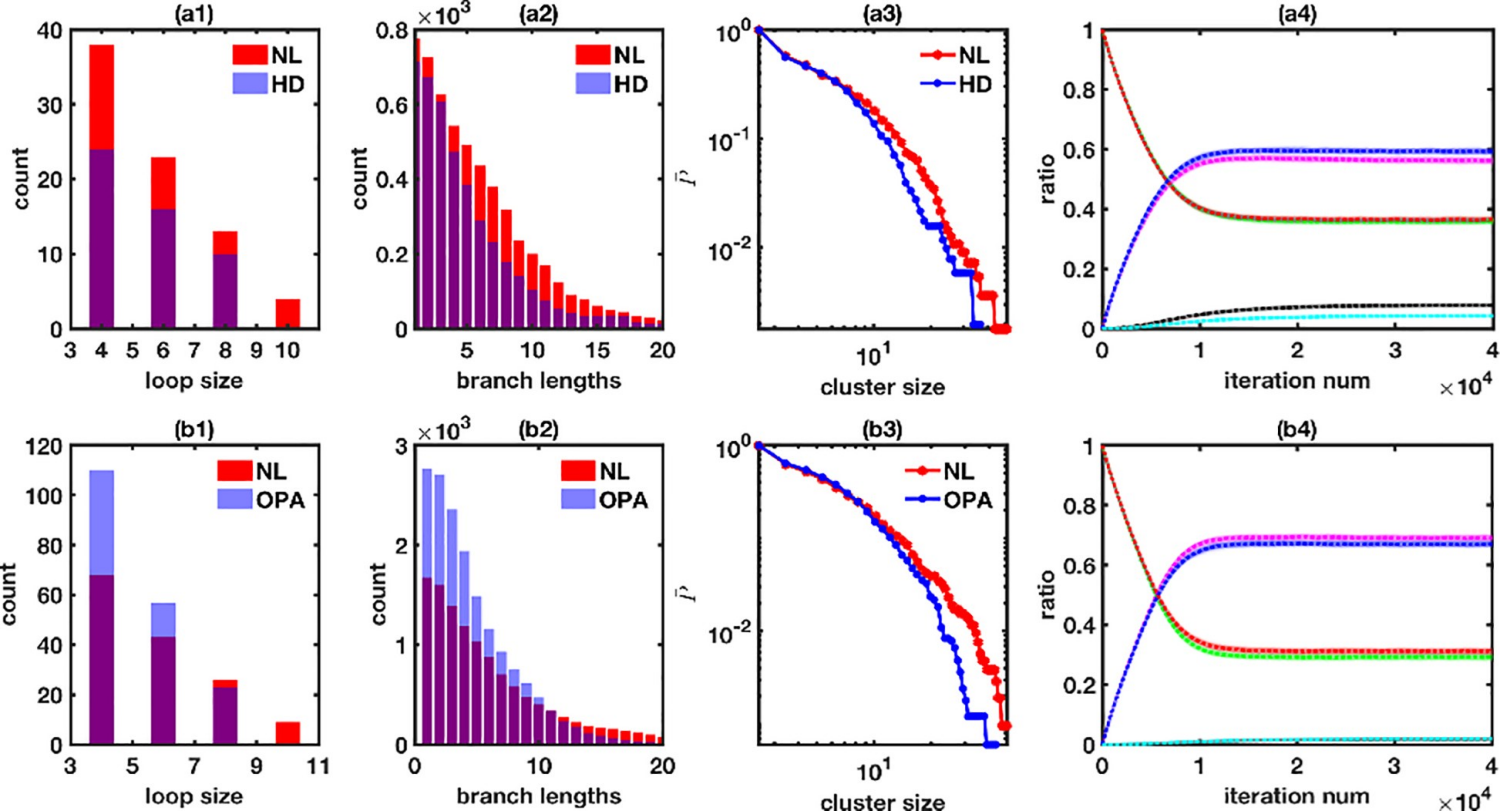
Two different types of microscopic changes in the fusion to fission processes leading to mitochondrial network fragmentation demonstrated with examples from HD (striatal cells from mouse embryos bearing a 111 polyglutamine repeat Q111/0 and Q111/1) versus control [78] for the first type (top row) and OPA (mouse embryonic fibroblasts with the pathogenic mutation R149W in human YME1L1) versus control [66] for the second type (bottom row) of microscopic changes. Distributions of (a1) loop sizes, (a2) branch lengths, and (a3) cluster sizes (cumulative probability) for NL (red) and diseased cells (blue) from experimental images. (a4) Fraction of X1 (NL: green, diseased: red), X2 (NL: magenta, diseased: blue) and X3 (NL: black, diseased: cyan) species from the model as functions of the number of iterations using C1 and C2 values for HD experiments. The model results show average of 100 runs. (b1-b4) shows the same mitochondrial network features as (a1-a4) for mouse embryonic fibroblasts with OPA pathology and their normal counterparts. Note that the curves for X3 species in cells with OPA pathology and NL overlap (b4).

A close look at the values of C1 and C2 reveals two main trends (Table 3). In case of HD, AD, DS, Ca^2+^ overload, kidney disease, and diabetes/cancer the fusion to fission ratio for the tip-to-tip reaction remains constant, while the fusion to fission ratio for the tip-to-side reaction decreases when compared to the control conditions. As shown by an example from HD (Fig 4A1–4A4), this results in smaller number of X3 species with a gain in X1 and X2 species in the diseased state (Fig 4A4 and Table 3). However, since the probability of cyclic loops depends on both X2 and X3, the large decrease in X3 and moderate increase in X2 lead to smaller cyclic loops and consequently smaller clusters in the diseased state (Fig 4A1 & 4A3). Larger number of X2 species with no change in X1 would translate into longer and/or larger number of linear branches. However, the simultaneous increase in the number of X1 species would result in shorter branches (Fig 4A2) and higher number of isolated mitochondria. A relatively smaller decrease in C2 leads to a smaller decrease in X3, and a smaller increase in X1 and X2, which would lead to smaller but larger number of linear chains. The larger number of linear chains could overcompensate for the small decrease in X3, resulting in a larger number of cyclic loops. Such behavior is demonstrated by an example using network statistics for diabetes (S2 Fig).

**Table 3:** Comparison of the fusion to fission ratio for the tip-to-tip and tip-to-side reactions in the normal and diseased states predicted by the model. The subscripts n and d indicate normal and diseased states respectively. The C1 and C2 values estimated for different conditions are used to estimate the fractions of X1, X2, and X3 species in steady state and compare them with the diseased states.

Condition	C_1n_/C_1d_	C_2n_/C_2d_	X_1n_	X_2n_	X_3n_	X_1n_/X_1d_	X_2n_/X_2d_	X_3n_/X_3d_
HD	1.00	2.00	0.359	0.562	0.079	0.985	0.948	1.852
AD	1.00	1.21	0.432	0.429	0.140	0.992	0.969	1.143
DS	1.00	2.75	0.454	0.502	0.044	0.980	0.939	17.058
CA	1.00	1.29	0.441	0.459	0.100	0.990	0.969	1.238
Kidney	1.01	1.60	0.344	0.605	0.052	0.991	0.971	1.704
Diabetes/ Cancer	1.00	1.80	0.334	0.614	0.052	0.990	0.971	1.739
OPA	1.18	1.00	0.292	0.691	0.018	0.938	1.030	0.955
PD	1.22	1.00	0.260	0.728	0.012	0.922	1.032	0.954
ALS	4.80	1.00	0.341	0.468	0.191	0.875	1.129	0.976

An opposite effect can be seen in case of OPA, PD, and ALS where C1 decreases and C2 remains constant when compared to normal cells. The lower fusion to fission ratio for the tip-to-tip reaction leads to larger and smaller number of X1 and X2 mitochondrial species respectively (Table 3). A larger decrease in C1 would lead to a larger increase in X1 and a larger decrease in X2, and consequently shorter, fewer linear chains (and larger number of isolated mitochondria) and vice versa. For example, the relatively smaller decrease in C1 in case of OPA leads to shorter linear branches (leftward shift in Fig 4B2) but the number of branches increases (taller bars) as compared to control conditions. Although the fusion to fission ratio for the tip-to-side reaction does not change, the larger number of linear chains available to make cyclic loops leads to a larger number of smaller loops (Fig 4B1). If the decrease in C1 is larger, one would see a significant decrease in the number of loops and branches (and significant increase in the number of isolated mitochondria) in addition to the leftward shift in the diseased case. Such behavior is demonstrated by an example using network statistics for PD (S3 Fig).

To see if the conclusions made above for a given disease holds when images of mitochondrial networks recorded from different cell/animal models or different experimental setup are used, we analyzed two more examples each for AD [121], PD [122], and ALS [91]. As clear from S2 Table, the results are largely consistent with our conclusions discussed above. The mean degree is higher for mitochondrial networks in NL cells as compared to their diseased counterparts. The microscopic rates (C2/C1) given by the simulations are also consistent with the above conclusions. With the exception of one example for AD and PD each, the normalized giant cluster (N_g_/N) for all cases from our simulations also follows a consistent trend. For the two examples where N_g_/N is slightly larger for NL cells than the diseased cells, we noticed that the overall mitochondrial network (network size in terms of the total number of edges in the cell) in the imaged area of the NL cells were significantly larger than those in the diseased cells. We suspect that this contributed to this discrepancy. Nevertheless, the mean degree in the same two examples is still consistent with our conclusions in the preceding paragraphs.

Despite the fact that the overall cumulative probability of the cluster sizes shifts to the left in all cases (see for example Fig 4A3 & 4B3), the different microscopic mechanisms for fragmentation lead to mitochondrial networks with significantly different fine structures. This is demonstrated by the fraction of X1, X2, and X3 species at steady state (Table 3, columns 4–9) obtained from simulations using C1 and C2 values for mitochondrial networks in cells with different pathologies and their respective control conditions. In the first group of conditions described above, the fraction of X3 species decreases significantly while X1 and X2 both increase moderately in the diseased state. This would lead to smaller and fewer cyclic loops. In the second group of diseases, X1 increases significantly while X2 decreases moderately. Since the propensity of X1+X2 → X3 reaction is given by a1 × X1 × X2, the relatively larger increase in X1 with the moderate decrease in X2 leads to a larger fraction of X3 species in the diseased state. A larger increase in X3 and a smaller decrease in X2 would lead to a larger number of cyclic loops (although still smaller in sizes) and vice versa.

**Fig 5:**
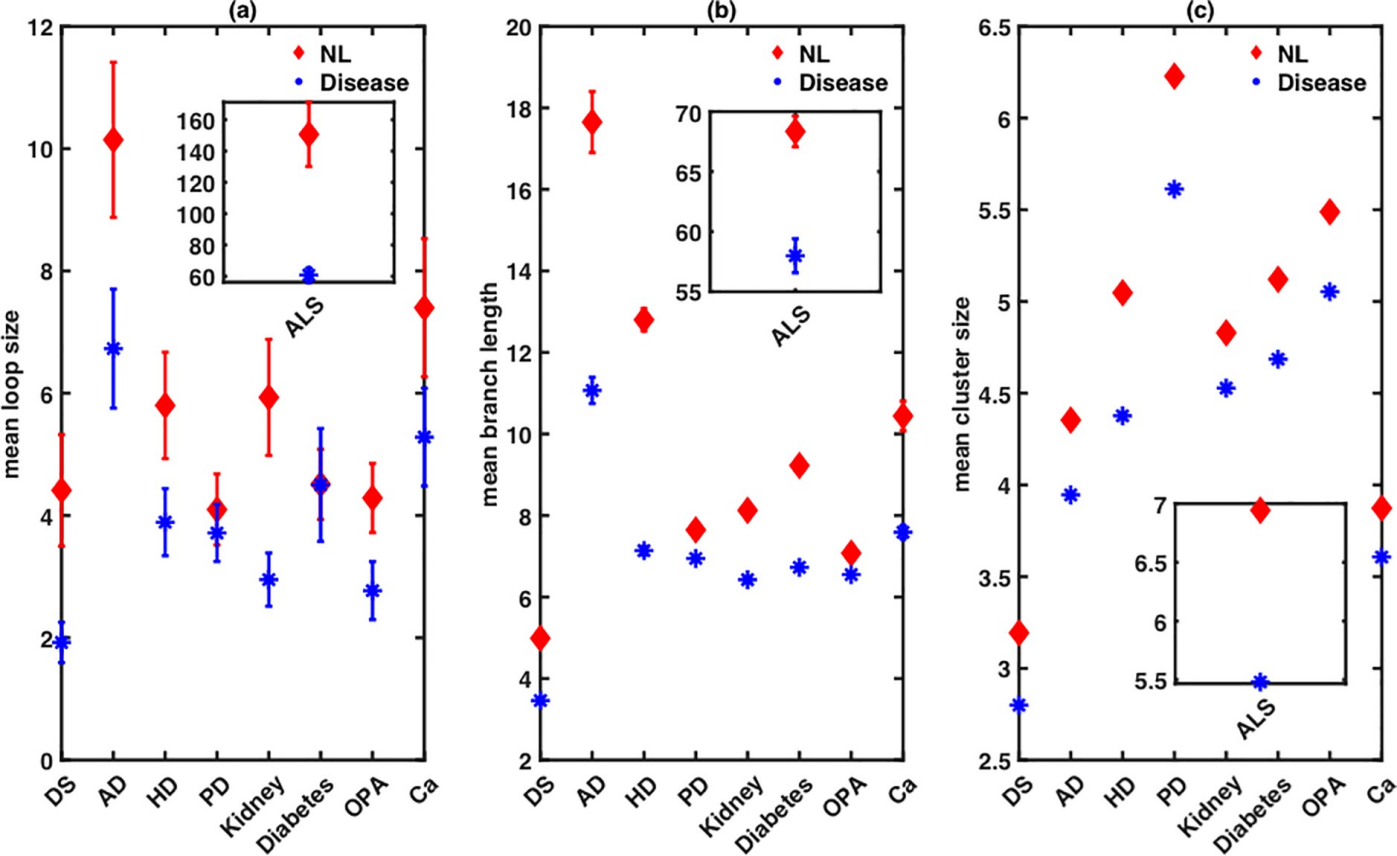
The differences in the microscopic changes leading to mitochondrial network fragmentation lead to significantly differences in the way the fine structure and topology of the network is affected in different diseases. The mean of size distribution of (a) cyclic loops, (b) branch lengths, and (c) clusters for normal (red) and diseased (blue) cells given by the model using the estimated C1 and C2 values from the experimental micrographs of mitochondrial networks with the condition modeled. Each data point is averaged over 100 runs with error bars showing the standard error of the mean. Simulation results for ALS are plotted separately in the insets for clarity.

The large variability in the fine structure of the mitochondrial network resulting from the different microscopic origins of fragmentation is highlighted further in Fig 5. We simulate mitochondrial networks in different diseases and their respective control conditions using their corresponding C1 and C2 values in the model, and extract the size distributions for branch lengths, cyclic loops, and clusters. The means of these distributions are shown in Fig 5, where the relative decrease vary significantly from one disease to another. A similar variability can also be seen in the variances of these three distributions while comparing different diseases to their respective control conditions (not shown).

## Discussion

A tight balance between fission and fusion of mitochondria is crucial for the normal cell function [20, 29, 123]. This is probably why many degenerative diseases have been linked to the primary or secondary changes in mitochondrial dynamics leading to fragmented mitochondrial networks [9, 15–36]. Our analysis of images of mitochondrial networks from several previously reported experimental studies indicates that in general mitochondria in normal cells form a well-connected network that can be described by larger mean degree, giant cluster, branch lengths, clusters, and loops as compared to fragmented network characterized by smaller values of all these parameters in cells with nine different types of pathologies. We exploit these differences and model mitochondrial network to gain a quantitative understanding of the changes in the fission and fusion processes due to lateral and longitudinal interactions in all these pathologies.

It is worth mentioning that the class (transient versus complete) of fusion depends on the way two mitochondria interact with each other (see for example [83] for further details). Transient fusion where two mitochondria come into close apposition, remain fused for less than 4s to 5 min with a mean duration of 45s, and re-separate, preserving their original topologies, results from oblique or lateral interaction of two mitochondria associated with separate tracks. Complete fusion on the other hand results from longitudinal merging of organelles moving along a single track.

We show that the nine conditions can be divided into two main groups. The fragmentation in cells with AD, HD, DS, Ca^2+^ overload, diabetes/cancer, and acute kidney injury pathologies mainly results from the decreased fusion in favor of fission due to lateral interaction between mitochondria. In case of OPA, PD, and ALS on the other hand, the balance between fusion and fission due to lateral interaction remains intact. However, the increased fission at the expense of fusion due to longitudinal interaction leads to fragmented mitochondrial network in these diseases.

The differences in the microscopic properties of mitochondrial fission and fusion could have key implications for the way fragmentation affects cell function depending on the morphology and the region of the cell where fragmentation occurs. For example, impaired balance between fission and fusion due to longitudinal interaction would lead to shorter linear chains of mitochondria that could significantly affect signaling along neuronal processes and synapses. Increased rate of fission at the expense of fusion due to lateral interaction on the other hand would likely have a more significant effect on the functions in regions such as cell body where a healthy mitochondrial network is key for the function of organelles such as nucleus and Golgi network.

We remark that our conclusions are based on limited data available. Consolidating these conclusions will need further future experiments and analysis of the mitochondrial networks in the different diseases using the approach discussed in this paper. Nevertheless, we believe that our framework provides a solid foundation for developing computational tools that could use these indicators for inferring the extent and types of signaling disruptions in different pathologies. While beyond the scope of this study, we believe that validating our predictions about the disruption of lateral and/or longitudinal fission/fusion in different diseases, experimental techniques similar to that used in Ref. [83] could be useful. In this technique, the exchange of matrix contents between individual mitochondria is visualized in real time as the two mitochondria fuse or detach by using mitochondrial matrix-targeted green-photoactivated, red-fluorescent Kindling fluorescent protein in combination with green or yellow fluorescence protein or the cyan-photoactivated mtPAGEP (mitochondria-targeted photoactivatable green-fluorescence protein) in combination with red fluorescence protein [83].

## Supporting information

S1 Text Description of different types of mitochondrial interactions, cell models, and diseases investigated in this study. (DOCX)

S1 Fig Longitudinal and lateral mitochondrial interactions (fusion/fission). (a) End-to-end fusion of two mitochondria moving towards each other along a common microtubule (not shown), (b) Side-to-side and end-to-side fusion of two mitochondria moving on two different microtubule tracks (not shown). Arrows indicate the direction of motion.
(TIFF)

S2 Fig A smaller decrease in C2 leads to a smaller decrease in X3, and a smaller increase in X1 and X2, which would lead to a smaller but larger number of linear chains and larger number of cyclic loops. Here we compare mitochondrial network fragmentation in HD (striatal cells from mouse embryos bearing a 111 polyglutamine repeat Q111/0 and Q111/1) versus control [78] with C_2n_/C_2d_ = 2.0 (top row) and diabetes (MitoNEET knockout mouse embryonic fibroblasts) versus control [65] with C_2n_/C_2d_ = 1.8 (bottom row). Distributions of (a1) loop sizes, (a2) branch lengths, and (a3) cluster sizes (cumulative probability) for NL (red) and diseased cells (blue) from experimental images. (a4) Fraction of X1 (NL: green, diseased: red), X2 (NL: magenta, diseased: blue) and X3 (NL: black, diseased: cyan) species from the model as functions of the number of iterations using C1 and C2 values for HD experiments. The model results show average of 100 runs. (b1-b4) shows the same mitochondrial network features as (a1-a4) for MitoNEET knockout mouse embryonic fibroblasts with diabetes pathology and their normal counterparts.
(TIFF)

S3 Fig A larger decrease in C1 leads to a significant decrease in the number of loops and branches. Here we compare mitochondrial network fragmentation in OPA (mouse embryonic fibroblasts with the pathogenic mutation R149W in human YME1L1) versus control [66] with C_1n_/C_1d_ = 1.18 (top row) and PD (human primary skin fibroblasts obtained from sporadic late-onset PD patients) versus those from healthy age-matched control subjects [81] with C_1n_/C_1d_ = 1.22 (bottom row). Distributions of (a1) loop sizes, (a2) branch lengths, and (a3) cluster sizes (cumulative probability) for NL (red) and diseased cells (blue) from experimental images. (a4) Fraction of X1 (NL: green, diseased: red), X2 (NL: magenta, diseased: blue) and X3 (NL: black, diseased: cyan) species from the model as functions of the number of iterations using C1 and C2 values for OPA experiments. The model results show average of 100 runs. (b1-b4) shows the same mitochondrial network features as (a1-a4) for human primary skin fibroblasts with PD pathology and their normal counterparts. Note that the curves for X3 species in diseased and normal cells overlap (a4, b4).
(TIFF)

S1 Table Experimental micrographs processed in this study. Column 1 provides the disease, column 3 reports the cell/animal model, column 4 lists the condition for the experiment (normal versus diseased), and column 5 provides references where the images were originally published.
**Abbreviations:** WT-Wild type, KO-Knockout, NL-Normal, MEF-Mouse embryonic fibroblasts, HSF-Human skin fibroblasts, MEMN Mouse embryonic motor neurons, TM-YAC128 Transgenic mice Yeast Artificial Chromosome 128, HSF Human Skin Fibroblasts, RPTCs rat proximal tubular cells, RCA rat cortical astrocytes, CHCHD10—Coiled-coil-helix-coiled-coil-helix domain-containing protein 10, YME1L1—Yeast mitochondrial escape 1-like 1 gene.
(DOCX)

S2 Table Comparison of microscopic parameters of mitochondrial network obtained from simulations and experiments for additional cell/animal models or experimental conditions on AD, PD, and ALS diseases. Column 1 lists the condition for which images of normal (NL) and diseased cells were analyzed (column 2). Columns 3 & 4 and 5 & 6 compare <k> and N_g_/N respectively from experiment and theory. Columns 7 & 8 are the C1 (tip-to-tip fusion/fission) and C2 (tip-to-side fusion/fission) values obtained by fitting the model to the data and used in simulations.
(DOCX)

## References

[1] Bakeeva L, Chentsov YS, Skulachev V. Mitochondrial framework (reticulum mitochondriale) in rat diaphragm muscle. Biochimica et Biophysica Acta (BBA)-Bioenergetics. 1978;501(3):349–69. 629958 10.1016/0005-2728(78)90104-4

[2] Amchenkova AA, Bakeeva LE, Chentsov YS, Skulachev VP, Zorov DB. Coupling membranes as energy-transmitting cables. I. Filamentous mitochondria in fibroblasts and mitochondrial clusters in cardiomyocytes. The Journal of cell biology. 1988;107(2):481–95. 10.1083/jcb.107.2.481 3417757 PMC2115217

[3] Szabadkai G, Simoni AM, Rizzuto R. Mitochondrial Ca2+ uptake requires sustained Ca2+ release from the endoplasmic reticulum. Journal of Biological Chemistry. 2003;278(17):15153–61. 10.1074/jbc.M300180200 12586823

[4] Anesti V, Scorrano L. The relationship between mitochondrial shape and function and the cytoskeleton. Biochimica et Biophysica Acta (BBA)-Bioenergetics. 2006;1757(5–6):692–9. 16729962 10.1016/j.bbabio.2006.04.013

[5] Yang J-S, Kim J, Park S, Jeon J, Shin Y-E, Kim S. Spatial and functional organization of mitochondrial protein network. Scientific reports. 2013;3:1403 10.1038/srep01403 23466738 PMC3590558

[6] Collins TJ, Berridge MJ, Lipp P, Bootman MD. Mitochondria are morphologically and functionally heterogeneous within cells. Embo Journal. 2002;21(7):1616–27. 10.1093/emboj/21.7.1616 WOS:000174992000012. 11927546 PMC125942

[7] Collins TJ, Lipp P, Berridge MJ, Bootman MD. Mitochondria are morphologically and functionally heterogeneous within single cells. Journal of Physiology-London. 2002;539:98p–9p. WOS:000174618200135. 10.1093/emboj/21.7.1616 PMC125942 11927546

[8] Bereiterhahn J, Voth M. Dynamics of Mitochondria in Living Cells—Shape Changes, Dislocations, Fusion, and Fission of Mitochondria. Microscopy Research and Technique. 1994;27(3):198–219. 10.1002/jemt.1070270303 WOS:A1994MV92300002. 8204911

[9] Karbowski M, Youle R. Dynamics of mitochondrial morphology in healthy cells and during apoptosis. Cell death and differentiation. 2003;10(8):870 10.1038/sj.cdd.4401260 12867994

[10] Detmer SA, Chan DC. Functions and dysfunctions of mitochondrial dynamics. Nature reviews Molecular cell biology. 2007;8(11):870 10.1038/nrm2275 17928812

[11] Benard G, Bellance N, James D, Parrone P, Fernandez H, Letellier T, et al Mitochondrial bioenergetics and structural network organization. Journal of cell science. 2007;120(5):838–48. 17298981 10.1242/jcs.03381

[12] Liao P-C, Tandarich LC, Hollenbeck PJ. ROS regulation of axonal mitochondrial transport is mediated by Ca2+ and JNK in Drosophila. PloS one. 2017;12(5):e0178105 10.1371/journal.pone.0178105 28542430 PMC5436889

[13] Debattisti V, Gerencser AA, Saotome M, Das S, Hajnóczky G. ROS control mitochondrial motility through p38 and the motor adaptor Miro/Trak. Cell reports. 2017;21(6):1667–80. 10.1016/j.celrep.2017.10.060 29117569 PMC5710826

[14] Deheshi S, Dabiri B, Fan S, Tsang M, Rintoul GL. Changes in mitochondrial morphology induced by calcium or rotenone in primary astrocytes occur predominantly through ros-mediated remodeling. Journal of Neurochemistry. 2015;133(5):684–99. 10.1111/jnc.13090 WOS:000353570500007. 25761412

[15] Schon EA, Przedborski S. Mitochondria: the next (neurode) generation. Neuron. 2011;70(6):1033–53. 10.1016/j.neuron.2011.06.003 21689593 PMC3407575

[16] Smith EF, Shaw PJ, De Vos KJ. The role of mitochondria in amyotrophic lateral sclerosis. Neuroscience letters. 2017. 10.1016/j.neulet.2017.06.052 28669745

[17] Guardia‐Laguarta C, Area‐Gomez E, Schon EA, Przedborski S. A new role for α‐synuclein in Parkinson's disease: Alteration of ER–mitochondrial communication. Movement Disorders. 2015;30(8):1026–33. 10.1002/mds.26239 25952565

[18] Eisner V, Picard M, Hajnóczky G. Mitochondrial dynamics in adaptive and maladaptive cellular stress responses. Nature cell biology. 2018:1 10.1038/s41556-017-0025-8 29950571 PMC6716149

[19] Bertholet A, Delerue T, Millet A, Moulis M, David C, Daloyau M, et al Mitochondrial fusion/fission dynamics in neurodegeneration and neuronal plasticity. Neurobiology of disease. 2016;90:3–19. 10.1016/j.nbd.2015.10.011 26494254

[20] Knott AB, Perkins G, Schwarzenbacher R, Bossy-Wetzel E. Mitochondrial fragmentation in neurodegeneration. Nature Reviews Neuroscience. 2008;9(7):505 10.1038/nrn2417 18568013 PMC2711514

[21] Chen H, McCaffery JM, Chan DC. Mitochondrial fusion protects against neurodegeneration in the cerebellum. Cell. 2007;130(3):548–62. 10.1016/j.cell.2007.06.026 17693261

[22] Hung CH-L, Cheng SS-Y, Cheung Y-T, Wuwongse S, Zhang NQ, Ho Y-S, et al A reciprocal relationship between reactive oxygen species and mitochondrial dynamics in neurodegeneration. Redox biology. 2018;14:7–19. 10.1016/j.redox.2017.08.010 28837882 PMC5567977

[23] Youle RJ, Karbowski M. Mitochondrial fission in apoptosis. Nature reviews Molecular cell biology. 2005;6(8):657 10.1038/nrm1697 16025099

[24] Perfettini J-L, Roumier T, Kroemer G. Mitochondrial fusion and fission in the control of apoptosis. Trends in cell biology. 2005;15(4):179–83. 10.1016/j.tcb.2005.02.005 15817372

[25] Manczak M, Calkins MJ, Reddy PH. Impaired mitochondrial dynamics and abnormal interaction of amyloid beta with mitochondrial protein Drp1 in neurons from patients with Alzheimer's disease: implications for neuronal damage. Human molecular genetics. 2011;20(13):2495–509. 10.1093/hmg/ddr139 21459773 PMC3109997

[26] Wang X, Su B, Fujioka H, Zhu X. Dynamin-like protein 1 reduction underlies mitochondrial morphology and distribution abnormalities in fibroblasts from sporadic Alzheimer's disease patients. The American journal of pathology. 2008;173(2):470–82. 10.2353/ajpath.2008.071208 18599615 PMC2475784

[27] Wang X, Su B, Siedlak SL, Moreira PI, Fujioka H, Wang Y, et al Amyloid-β overproduction causes abnormal mitochondrial dynamics via differential modulation of mitochondrial fission/fusion proteins. Proceedings of the National Academy of Sciences. 2008;105(49):19318–23. 10.1073/pnas.0804871105 PMC2614759 19050078

[28] Wang X, Su B, Lee H-g, Li X, Perry G, Smith MA, et al Impaired balance of mitochondrial fission and fusion in Alzheimer's disease. Journal of Neuroscience. 2009;29(28):9090–103. 10.1523/JNEUROSCI.1357-09.2009 19605646 PMC2735241

[29] Selfridge JE, Lezi E, Lu J, Swerdlow RH. Role of mitochondrial homeostasis and dynamics in Alzheimer's disease. Neurobiology of disease. 2013;51:3–12. 10.1016/j.nbd.2011.12.057 22266017 PMC3337963

[30] Hedskog L, Pinho CM, Filadi R, Rönnbäck A, Hertwig L, Wiehager B, et al Modulation of the endoplasmic reticulum–mitochondria interface in Alzheimer’s disease and related models. Proceedings of the National Academy of Sciences. 2013:201300677. 10.1073/pnas.1300677110 PMC3651455 23620518

[31] Area-Gomez E, Schon EA. On the pathogenesis of Alzheimer's disease: the MAM hypothesis. The FASEB Journal. 2017;31(3):864–7. 10.1096/fj.201601309 28246299 PMC6191063

[32] Aon MA, Cortassa S, Akar FG, Brown DA, Zhou L, O'Rourke B. From mitochondrial dynamics to arrhythmias. International Journal of Biochemistry & Cell Biology. 2009;41(10):1940–8. 10.1016/j.biocel.2009.02.016 WOS:000270351100021. 19703656 PMC2732583

[33] Grandemange S, Herzig S, Martinou JC. Mitochondrial dynamics and cancer. Seminars in Cancer Biology. 2009;19(1):50–6. 10.1016/j.semcancer.2008.12.001 WOS:000264608700008. 19138741

[34] Su B, Wang XL, Zheng L, Perry G, Smith MA, Zhu XW. Abnormal mitochondrial dynamics and neurodegenerative diseases. Biochimica Et Biophysica Acta-Molecular Basis of Disease. 2010;1802(1):135–42. 10.1016/j.bbadis.2009.09.013 WOS:000273138500015. 19799998 PMC2790543

[35] Yoon Y, Galloway CA, Jhun BS, Yu TZ. Mitochondrial Dynamics in Diabetes. Antioxidants & Redox Signaling. 2011;14(3):439–57. 10.1089/ars.2010.3286 WOS:000285876900010. 20518704 PMC3025181

[36] Zamponi E, Zamponi N, Coskun P, Quassollo G, Lorenzo A, Cannas SA, et al Nrf2 stabilization prevents critical oxidative damage in Down syndrome cells. Aging Cell. 2018;17(5). UNSP e12812 10.1111/acel.12812 WOS:000445599100008. PMC6156351 30028071

[37] Izzo A, Mollo N, Nitti M, Paladino S, Calì G, Genesio R, et al Mitochondrial dysfunction in down syndrome: molecular mechanisms and therapeutic targets. Molecular Medicine. 2018;24(1):2 10.1186/s10020-018-0004-y 30134785 PMC6016872

[38] Kann O, Kovács R. Mitochondria and neuronal activity. American Journal of Physiology-Cell Physiology. 2007;292(2):C641–C57. 10.1152/ajpcell.00222.2006 17092996

[39] Li Z, Okamoto K-I, Hayashi Y, Sheng M. The importance of dendritic mitochondria in the morphogenesis and plasticity of spines and synapses. Cell. 2004;119(6):873–87. 10.1016/j.cell.2004.11.003 15607982

[40] Lin MT, Beal MF. Mitochondrial dysfunction and oxidative stress in neurodegenerative diseases. Nature. 2006;443(7113):787 10.1038/nature05292 17051205

[41] Sheng Z-H, Cai Q. Mitochondrial transport in neurons: impact on synaptic homeostasis and neurodegeneration. Nature Reviews Neuroscience. 2012;13(2):77 10.1038/nrn3156 22218207 PMC4962561

[42] Westermann B. Bioenergetic role of mitochondrial fusion and fission. Biochimica et Biophysica Acta (BBA)-Bioenergetics. 2012;1817(10):1833–8. 22409868 10.1016/j.bbabio.2012.02.033

[43] Bach D, Pich S, Soriano FX, Vega N, Baumgartner B, Oriola J, et al Mitofusin-2 determines mitochondrial network architecture and mitochondrial metabolism: a novel regulatory mechanism altered in obesity. Journal of Biological Chemistry. 2003. 10.1074/jbc.M212754200 12598526

[44] Olichon A, Baricault L, Gas N, Guillou E, Valette A, Belenguer P, et al Loss of OPA1 perturbates the mitochondrial inner membrane structure and integrity, leading to cytochrome c release and apoptosis. Journal of Biological Chemistry. 2003;278(10):7743–6. 10.1074/jbc.C200677200 12509422

[45] Chen H, Chomyn A, Chan DC. Disruption of fusion results in mitochondrial heterogeneity and dysfunction. Journal of Biological Chemistry. 2005;280(28):26185–92. 10.1074/jbc.M503062200 15899901

[46] Benard G, Rossignol R. Ultrastructure of the mitochondrion and its bearing on function and bioenergetics. Antioxidants & redox signaling. 2008;10(8):1313–42. 18435594 10.1089/ars.2007.2000

[47] Cheung EC, McBride HM, Slack RS. Mitochondrial dynamics in the regulation of neuronal cell death. Apoptosis. 2007;12(5):979–92. 10.1007/s10495-007-0745-5 17453163

[48] Jahani‐Asl A, Slack RS. The phosphorylation state of Drp1 determines cell fate. EMBO reports. 2007;8(10):912–3. 10.1038/sj.embor.7401077 17906671 PMC2002565

[49] Chen H, Chan DC. Mitochondrial dynamics–fusion, fission, movement, and mitophagy–in neurodegenerative diseases. Human molecular genetics. 2009;18(R2):R169–R76. 10.1093/hmg/ddp326 19808793 PMC2758711

[50] Capaldi RA, Murray J, Byrne L, Janes MS, Marusich MF. Immunological approaches to the characterization and diagnosis of mitochondrial disease. Mitochondrion. 2004;4(5):417–26. 16120403 10.1016/j.mito.2004.07.006

[51] Koopman WJ, Visch H-J, Verkaart S, van den Heuvel LW, Smeitink JA, Willems PH. Mitochondrial network complexity and pathological decrease in complex I activity are tightly correlated in isolated human complex I deficiency. American Journal of Physiology-Cell Physiology. 2005;289(4):C881–C90. 10.1152/ajpcell.00104.2005 15901599

[52] Yu T, Robotham JL, Yoon Y. Increased production of reactive oxygen species in hyperglycemic conditions requires dynamic change of mitochondrial morphology. Proceedings of the National Academy of Sciences. 2006;103(8):2653–8. 10.1073/pnas.0511154103 PMC1413838 16477035

[53] Szabadkai G, Simoni AM, Chami M, Wieckowski MR, Youle RJ, Rizzuto R. Drp-1-dependent division of the mitochondrial network blocks intraorganellar Ca2+ waves and protects against Ca2+-mediated apoptosis. Molecular cell. 2004;16(1):59–68. 10.1016/j.molcel.2004.09.026 15469822

[54] Frieden M, James D, Castelbou C, Danckaert A, Martinou J-C, Demaurex N. Calcium homeostasis during mitochondria fragmentation and perinuclear clustering induced by hFis1. Journal of Biological Chemistry. 2004. 10.1074/jbc.M312366200 15024001

[55] Fang C, Bourdette D, Banker G. Oxidative stress inhibits axonal transport: implications for neurodegenerative diseases. Molecular neurodegeneration. 2012;7(1):29. 22709375 10.1186/1750-1326-7-29 PMC3407799

[56] Deheshi S, Dabiri B, Fan S, Tsang M, Rintoul GL. Changes in mitochondrial morphology induced by calcium or rotenone in primary astrocytes occur predominantly through ROS‐mediated remodeling. Journal of neurochemistry. 2015;133(5):684–99. 10.1111/jnc.13090 25761412

[57] Saotome M, Safiulina D, Szabadkai G, Das S, Fransson Å, Aspenstrom P, et al Bidirectional Ca2+-dependent control of mitochondrial dynamics by the Miro GTPase. Proceedings of the National Academy of Sciences. 2008;105(52):20728–33. 10.1073/pnas.0808953105 PMC2634948 19098100

[58] Jeyaraju DV, Cisbani G, Pellegrini L. Calcium regulation of mitochondria motility and morphology. Biochimica et Biophysica Acta (BBA)-Bioenergetics. 2009;1787(11):1363–73. 19138660 10.1016/j.bbabio.2008.12.005

[59] Youle RJ, Van Der Bliek AM. Mitochondrial fission, fusion, and stress. Science. 2012;337(6098):1062–5. 10.1126/science.1219855 22936770 PMC4762028

[60] Mishra P, Chan DC. Metabolic regulation of mitochondrial dynamics. J Cell Biol. 2016;212(4):379–87. 10.1083/jcb.201511036 26858267 PMC4754720

[61] Szabadkai G, Simoni A, Bianchi K, De Stefani D, Leo S, Wieckowski M, et al Mitochondrial dynamics and Ca2+ signaling. Biochimica et Biophysica Acta (BBA)-Molecular Cell Research. 2006;1763(5–6):442–9. 16750865 10.1016/j.bbamcr.2006.04.002

[62] Tan AR, Cai AY, Deheshi S, Rintoul GL. Elevated intracellular calcium causes distinct mitochondrial remodelling and calcineurin-dependent fission in astrocytes. Cell calcium. 2011;49(2):108–14. 10.1016/j.ceca.2010.12.002 21216007

[63] Liu X, Hajnóczky G. Ca2+-dependent regulation of mitochondrial dynamics by the Miro–Milton complex. The international journal of biochemistry & cell biology. 2009;41(10):1972–6. 19481172 10.1016/j.biocel.2009.05.013 PMC2742205

[64] Brooks C, Wei Q, Cho S-G, Dong Z. Regulation of mitochondrial dynamics in acute kidney injury in cell culture and rodent models. The Journal of clinical investigation. 2009;119(5):1275–85. 10.1172/JCI37829 19349686 PMC2673870

[65] Molina AJ, Wikstrom JD, Stiles L, Las G, Mohamed H, Elorza A, et al Mitochondrial networking protects beta cells from nutrient induced apoptosis. Diabetes. 2009. 10.2337/db07-1781 PMC2750232 19581419

[66] Hartmann B, Wai T, Hu H, MacVicar T, Musante L, Fischer-Zirnsak B, et al Homozygous YME1L1 mutation causes mitochondriopathy with optic atrophy and mitochondrial network fragmentation. Elife. 2016;5:e16078 10.7554/eLife.16078 27495975 PMC4991934

[67] Coskun PE, Busciglio J. Oxidative stress and mitochondrial dysfunction in Down’s syndrome: relevance to aging and dementia. Current gerontology and geriatrics research. 2012;2012. 10.1155/2012/383170 PMC3350950 22611387

[68] Helguera P, Seiglie J, Rodriguez J, Hanna M, Helguera G, Busciglio J. Adaptive downregulation of mitochondrial function in down syndrome. Cell metabolism. 2013;17(1):132–40. 10.1016/j.cmet.2012.12.005 23312288 PMC3580189

[69] Busciglio J, Yankner BA. Apoptosis and increased generation of reactive oxygen species in Down's syndrome neurons in vitro. Nature. 1995;378(6559):776 10.1038/378776a0 8524410

[70] Busciglio J, Pelsman A, Wong C, Pigino G, Yuan M, Mori H, et al Altered metabolism of the amyloid β precursor protein is associated with mitochondrial dysfunction in Down's syndrome. Neuron. 2002;33(5):677–88. 10.1016/s0896-6273(02)00604-9 11879646

[71] Peng J-Y, Lin C-C, Chen Y-J, Kao L-S, Liu Y-C, Chou C-C, et al Automatic morphological subtyping reveals new roles of caspases in mitochondrial dynamics. PLoS computational biology. 2011;7(10):e1002212 10.1371/journal.pcbi.1002212 21998575 PMC3188504

[72] J Tronstad K, Nooteboom M, IH Nilsson L, Nikolaisen J, Sokolewicz M, Grefte S, et al Regulation and quantification of cellular mitochondrial morphology and content. Current pharmaceutical design. 2014;20(35):5634–52. 10.2174/1381612820666140305230546 24606803

[73] Quirós PM, Ramsay AJ, Sala D, Fernández‐Vizarra E, Rodríguez F, Peinado JR, et al Loss of mitochondrial protease OMA1 alters processing of the GTPase OPA1 and causes obesity and defective thermogenesis in mice. The EMBO journal. 2012;31(9):2117–33. 10.1038/emboj.2012.70 22433842 PMC3343468

[74] Dirnberger M, Kehl T, Neumann A. NEFI: Network extraction from images. Scientific reports. 2015;5:15669 10.1038/srep15669 26521675 PMC4629128

[75] Zamponi N, Zamponi E, Cannas SA, Billoni OV, Helguera PR, Chialvo DR. Mitochondrial network complexity emerges from fission/fusion dynamics. Scientific Reports. 2018;8 ARTN 363 10.1038/s41598-017-18351-5 WOS:000419672300008. 29321534 PMC5762699

[76] Sukhorukov VM, Dikov D, Reichert AS, Meyer-Hermann M. Emergence of the Mitochondrial Reticulum from Fission and Fusion Dynamics. Plos Computational Biology. 2012;8(10). ARTN e1002745 10.1371/journal.pcbi.1002745 WOS:000310568800040. 23133350 PMC3486901

[77] Reis Y, Bernardo-Faura M, Richter D, Wolf T, Brors B, Hamacher-Brady A, et al Multi-parametric analysis and modeling of relationships between mitochondrial morphology and apoptosis. PLoS One. 2012;7(1):e28694 10.1371/journal.pone.0028694 22272225 PMC3260148

[78] Costa V, Giacomello M, Hudec R, Lopreiato R, Ermak G, Lim D, et al Mitochondrial fission and cristae disruption increase the response of cell models of Huntington's disease to apoptotic stimuli. EMBO molecular medicine. 2010;2(12):490–503. 10.1002/emmm.201000102 21069748 PMC3044888

[79] Wang XL, Su B, Siedlak SL, Moreira PI, Fujioka H, Wang Y, et al Amyloid-beta overproduction causes abnormal mitochondrial dynamics via differential modulation of mitochondrial fission/fusion proteins. Proceedings of the National Academy of Sciences of the United States of America. 2008;105(49):19318–23. 10.1073/pnas.0804871105 WOS:000261706600054. 19050078 PMC2614759

[80] Woo J-A, Liu T, Trotter C, Fang CC, De Narvaez E, LePochat P, et al Loss of function CHCHD10 mutations in cytoplasmic TDP-43 accumulation and synaptic integrity. Nature Communications. 2017;8:15558 10.1038/ncomms15558 28585542 PMC5467170

[81] Teves JM, Bhargava V, Kirwan KR, Corenblum MJ, Justiniano R, Wondrak GT, et al Parkinson's Disease Skin Fibroblasts Display Signature Alterations in Growth, Redox Homeostasis, Mitochondrial Function, and Autophagy. Frontiers in neuroscience. 2018;11:737 10.3389/fnins.2017.00737 29379409 PMC5770791

[82] Izzo A, Nitti M, Mollo N, Paladino S, Procaccini C, Faicchia D, et al Metformin restores the mitochondrial network and reverses mitochondrial dysfunction in Down syndrome cells. Human molecular genetics. 2017;26(6):1056–69. 10.1093/hmg/ddx016 28087733

[83] Liu X, Weaver D, Shirihai O, Hajnóczky G. Mitochondrial ‘kiss‐and‐run’: interplay between mitochondrial motility and fusion–fission dynamics. The EMBO journal. 2009;28(20):3074–89. 10.1038/emboj.2009.255 19745815 PMC2771091

[84] Gillespie DT. Exact Stochastic Simulation of Coupled Chemical-Reactions. Journal of Physical Chemistry. 1977;81(25):2340–61. 10.1021/j100540a008 WOS:A1977EE49800008.

[85] Costa RO, Ferreiro E, Cardoso SM, Oliveira CR, Pereira CM. ER stress-mediated apoptotic pathway induced by Aβ peptide requires the presence of functional mitochondria. Journal of Alzheimer's Disease. 2010;20(2):625–36. 10.3233/JAD-2010-091369 20182029

[86] Bannwarth S, Ait-El-Mkadem S, Chaussenot A, Genin EC, Lacas-Gervais S, Fragaki K, et al A mitochondrial origin for frontotemporal dementia and amyotrophic lateral sclerosis through CHCHD10 involvement. Brain. 2014;137(8):2329–45. 24934289 10.1093/brain/awu138 PMC4107737

[87] Zhang M, Xi Z, Zinman L, Bruni AC, Maletta RG, Curcio SA, et al Mutation analysis of CHCHD10 in different neurodegenerative diseases. Brain. 2015;138(9):e380–e. 25833818 10.1093/brain/awv082 PMC4547051

[88] Penttilä S, Jokela M, Bouquin H, Saukkonen AM, Toivanen J, Udd B. Late onset spinal motor neuronopathy is caused by mutation in CHCHD 10. Annals of neurology. 2015;77(1):163–72. 10.1002/ana.24319 25428574

[89] Auranen M, Ylikallio E, Shcherbii M, Paetau A, Kiuru-Enari S, Toppila JP, et al CHCHD10 variant p.(Gly66Val) causes axonal Charcot-Marie-Tooth disease. Neurology Genetics. 2015;1(1):e1 10.1212/NXG.0000000000000003 27066538 PMC4821082

[90] Xu Y-F, Gendron TF, Zhang Y-J, Lin W-L, D'Alton S, Sheng H, et al Wild-type human TDP-43 expression causes TDP-43 phosphorylation, mitochondrial aggregation, motor deficits, and early mortality in transgenic mice. Journal of Neuroscience. 2010;30(32):10851–9. 10.1523/JNEUROSCI.1630-10.2010 20702714 PMC3056148

[91] Wang W, Li L, Lin W-L, Dickson DW, Petrucelli L, Zhang T, et al The ALS disease-associated mutant TDP-43 impairs mitochondrial dynamics and function in motor neurons. Human molecular genetics. 2013;22(23):4706–19. 10.1093/hmg/ddt319 23827948 PMC3820133

[92] Janssens J, Van Broeckhoven C. Pathological mechanisms underlying TDP-43 driven neurodegeneration in FTLD–ALS spectrum disorders. Human molecular genetics. 2013;22(R1):R77–R87. 10.1093/hmg/ddt349 23900071 PMC3782069

[93] Buratti E. Functional significance of TDP-43 mutations in disease. Advances in genetics. 91: Elsevier; 2015 p. 1–53. 10.1016/bs.adgen.2015.07.001 26410029

[94] Josephs KA, Whitwell JL, Tosakulwong N, Weigand SD, Murray ME, Liesinger AM, et al TAR DNA‐binding protein 43 and pathological subtype of Alzheimer's disease impact clinical features. Annals of neurology. 2015;78(5):697–709. 10.1002/ana.24493 26224156 PMC4623932

[95] Wang W, Wang L, Lu J, Siedlak SL, Fujioka H, Liang J, et al The inhibition of TDP-43 mitochondrial localization blocks its neuronal toxicity. Nature medicine. 2016;22(8):869 10.1038/nm.4130 27348499 PMC4974139

[96] Zhang Y-J, Xu Y-F, Cook C, Gendron TF, Roettges P, Link CD, et al Aberrant cleavage of TDP-43 enhances aggregation and cellular toxicity. Proceedings of the National Academy of Sciences. 2009;106(18):7607–12. 10.1073/pnas.0900688106 PMC2671323 19383787

[97] Sohn Y-S, Tamir S, Song L, Michaeli D, Matouk I, Conlan AR, et al NAF-1 and mitoNEET are central to human breast cancer proliferation by maintaining mitochondrial homeostasis and promoting tumor growth. Proceedings of the National Academy of Sciences. 2013;110(36):14676–81. 10.1073/pnas.1313198110 PMC3767537 23959881

[98] Kusminski CM, Holland WL, Sun K, Park J, Spurgin SB, Lin Y, et al MitoNEET-driven alterations in adipocyte mitochondrial activity reveal a crucial adaptive process that preserves insulin sensitivity in obesity. Nature medicine. 2012;18(10):1539 10.1038/nm.2899 22961109 PMC3745511

[99] Kusminski CM, Chen S, Ye R, Sun K, Wang QA, Spurgin SB, et al MitoNEET-Parkin effects in pancreatic α-and β-cells, cellular survival, and intrainsular cross talk. Diabetes. 2016;65(6):1534–55. 10.2337/db15-1323 26895793 PMC5310214

[100] Geldenhuys WJ, Leeper TC, Carroll RT. mitoNEET as a novel drug target for mitochondrial dysfunction. Drug discovery today. 2014;19(10):1601–6. 10.1016/j.drudis.2014.05.001 24814435

[101] Vernay A, Marchetti A, Sabra A, Jauslin TN, Rosselin M, Scherer PE, et al MitoNEET-dependent formation of intermitochondrial junctions. Proceedings of the National Academy of Sciences. 2017;114(31):8277–82. 10.1073/pnas.1706643114 PMC5547643 28716905

[102] Finsterer J. Mitochondriopathies. European Journal of Neurology. 2004;11(3):163–86. 15009163 10.1046/j.1351-5101.2003.00728.x

[103] Rainbolt TK, Lebeau J, Puchades C, Wiseman RL. Reciprocal degradation of YME1L and OMA1 adapts mitochondrial proteolytic activity during stress. Cell reports. 2016;14(9):2041–9. 10.1016/j.celrep.2016.02.011 26923599 PMC4785047

[104] Anand R, Wai T, Baker MJ, Kladt N, Schauss AC, Rugarli E, et al The i-AAA protease YME1L and OMA1 cleave OPA1 to balance mitochondrial fusion and fission. J Cell Biol. 2014;204(6):919–29. 10.1083/jcb.201308006 24616225 PMC3998800

[105] Mishra P, Carelli V, Manfredi G, Chan DC. Proteolytic cleavage of Opa1 stimulates mitochondrial inner membrane fusion and couples fusion to oxidative phosphorylation. Cell metabolism. 2014;19(4):630–41. 10.1016/j.cmet.2014.03.011 24703695 PMC4018240

[106] Song Z, Chen H, Fiket M, Alexander C, Chan DC. OPA1 processing controls mitochondrial fusion and is regulated by mRNA splicing, membrane potential, and Yme1L. J Cell Biol. 2007;178(5):749–55. 10.1083/jcb.200704110 17709429 PMC2064540

[107] Berridge MJ. Calcium signalling remodelling and disease. Portland Press Limited; 2012. 10.1042/BST20110766 22435804

[108] Berridge MJ. Calcium signalling in health and disease. Biochemical and biophysical research communications. 2017;485(1):5–. 10.1016/j.bbrc.2017.01.098 28130105

[109] Bezprozvanny I. Calcium signaling and neurodegenerative diseases. Trends in molecular medicine. 2009;15(3):89–100. 10.1016/j.molmed.2009.01.001 19230774 PMC3226745

[110] Carafoli E, Brini M. Calcium signalling and disease: molecular pathology of calcium: Springer Science & Business Media; 2007.

[111] Berridge MJ, Lipp P, Bootman MD. The versatility and universality of calcium signalling. Nature reviews Molecular cell biology. 2000;1(1):11 10.1038/35036035 11413485

[112] Massry SG, Fadda GZ. Chronic renal failure is a state of cellular calcium toxicity. American journal of kidney diseases. 1993;21(1):81–6. 10.1016/s0272-6386(12)80727-x 8418632

[113] Rivera A, Conlin PR, Williams GH, Canessa ML. Elevated lymphocyte cytosolic calcium in a subgroup of essential hypertensive subjects. Hypertension. 1996;28(2):213–8. 10.1161/01.hyp.28.2.213 8707384

[114] Massry S, Smogorzewski M. Role of elevated cytosolic calcium in the pathogenesis of complications in diabetes mellitus. Mineral and electrolyte metabolism. 1997;23(3–6):253–60. 9387128

[115] Mattson MP, Chan SL. Neuronal and glial calcium signaling in Alzheimer’s disease. Cell calcium. 2003;34(4–5):385–97. 12909083 10.1016/s0143-4160(03)00128-3

[116] Lajdova I, Spustova V, Oksa A, Chorvatova A, Chorvat D Jr, Dzurik R. Intracellular calcium homeostasis in patients with early stagesof chronic kidney disease: effects of vitamin D3 supplementation. Nephrology Dialysis Transplantation. 2009;24(11):3376–81. 10.1093/ndt/gfp292 19531669

[117] HEATH H III, LAMBERT PW, SERVICE FJ, ARNAUD SB. Calcium homeostasis in diabetes mellitus. The Journal of Clinical Endocrinology & Metabolism. 1979;49(3):462–6. 468980 10.1210/jcem-49-3-462

[118] Ahn C, An B-S, Jeung E-B. Streptozotocin induces endoplasmic reticulum stress and apoptosis via disruption of calcium homeostasis in mouse pancreas. Molecular and cellular endocrinology. 2015;412:302–8. 10.1016/j.mce.2015.05.017 26003140

[119] Kushnareva Y, Gerencser A, Bossy B, Ju W, White A, Waggoner J, et al Loss of OPA1 disturbs cellular calcium homeostasis and sensitizes for excitotoxicity. Cell death and differentiation. 2013;20(2):353 10.1038/cdd.2012.128 23138851 PMC3554330

[120] Ahn C, Kang J-H, Jeung E-B. Calcium homeostasis in diabetes mellitus. Journal of veterinary science. 2017;18(3):261–6. 10.4142/jvs.2017.18.3.261 28927245 PMC5639077

[121] Pérez MJ, Ponce DP, Osorio-Fuentealba C, Behrens MI, Quintanilla RA. Mitochondrial bioenergetics is altered in fibroblasts from patients with sporadic Alzheimer's disease. Frontiers in neuroscience. 2017;11:553 10.3389/fnins.2017.00553 29056898 PMC5635042

[122] Krebiehl G, Ruckerbauer S, Burbulla LF, Kieper N, Maurer B, Waak J, et al Reduced basal autophagy and impaired mitochondrial dynamics due to loss of Parkinson's disease-associated protein DJ-1. PloS one. 2010;5(2):e9367 10.1371/journal.pone.0009367 20186336 PMC2826413

[123] Santel A. Get the balance right: mitofusins roles in health and disease. Biochimica et Biophysica Acta (BBA)-Molecular Cell Research. 2006;1763(5–6):490–9. 16574259 10.1016/j.bbamcr.2006.02.004

