# Supplementary material for: Mitochondrial fragmentation and network architecture in degenerative diseases
Source: PLoS One. 2019 Sep 26;14(9):e0223014. doi: 10.1371/journal.pone.0223014 (PMC6762132; doi:10.1371/journal.pone.0223014)
Supplement: S1 Text — (DOCX) [file pone.0223014.s001.docx]

**S1 Text**

**Mitochondrial Fragmentation and Network Architecture in Degenerative Diseases**

Syed I Shah, Johanna G Paine, Carlos Perez, and Ghanim Ullah

**Mitochondrial interactions**

Due to dynamic nature of mitochondria in terms of its microtubule-mediated movement throughout the cell to fulfill energy needs of various organelles of cell, mitochondria continually undergo fission and fusion mediated by the relevant proteins. These fission and fusion events have been classified into two broad categories, namely longitudinal and lateral interactions (S1 Fig). In the case of longitudinal interactions, two mitochondria moving in opposite directions along the same microtubule (axis) interact in an end-to-end fashion to form one mitochondrion (S1 Fig (a)). Generally, this type of interaction leads to a complete fusion of the two interacting mitochondria [1]. On the other hand, mitochondria on two different microtubules can interact through what is known as lateral interactions in two different ways, namely side-to-side and end-to-side as shown in S1 Fig (b). This type of fusion is relatively short-lived and the two mitochondria separate on seconds to minutes timescale after exchanging the matrix content.

**Micrographs processed**

Experimental micrographs processed in current study along with cell model (column 3), conditions of the experiment (column 4), and reference for each image (column 5) that are used in this study for various diseases are given in S1 Table.

**References**

1. Liu XG, Weaver D, Shirihai O, Hajnoczky G. Mitochondrial 'kiss-and-run': interplay between mitochondrial motility and fusion-fission dynamics. Embo J. 2009;28(20):3074-89. doi: 10.1038/emboj.2009.255. PubMed PMID: WOS:000271008200003.
