# Supplementary figures and images for: Mitochondrial fragmentation and network architecture in degenerative diseases
Source: PLoS One. 2019 Sep 26;14(9):e0223014. doi: 10.1371/journal.pone.0223014 (PMC6762132; doi:10.1371/journal.pone.0223014)

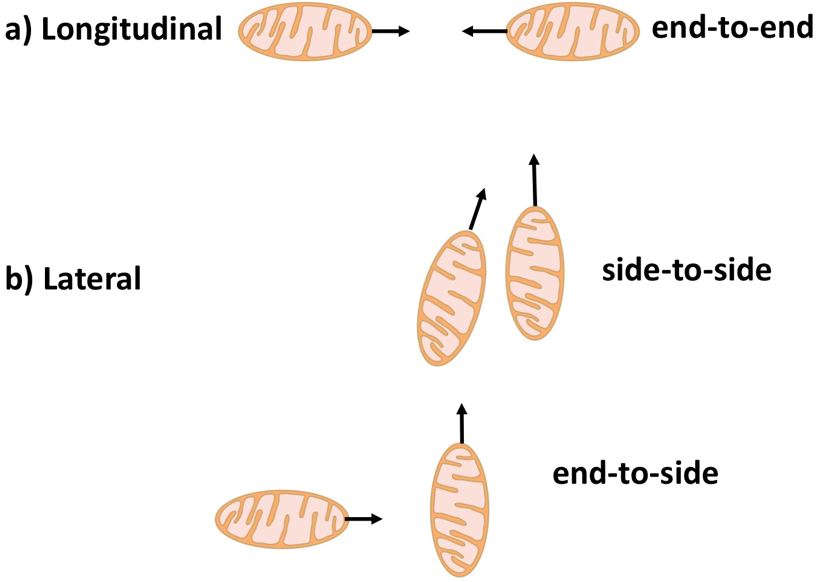

Supplement: S1 Fig — (a) End-to-end fusion of two mitochondria moving towards each other along a common microtubule (not shown), (b) Side-to-side and end-to-side fusion of two mitochondria moving on two different microtubule tracks (not shown). Arrows indicate the direction of motion. (TIFF) [file pone.0223014.s002.tiff]

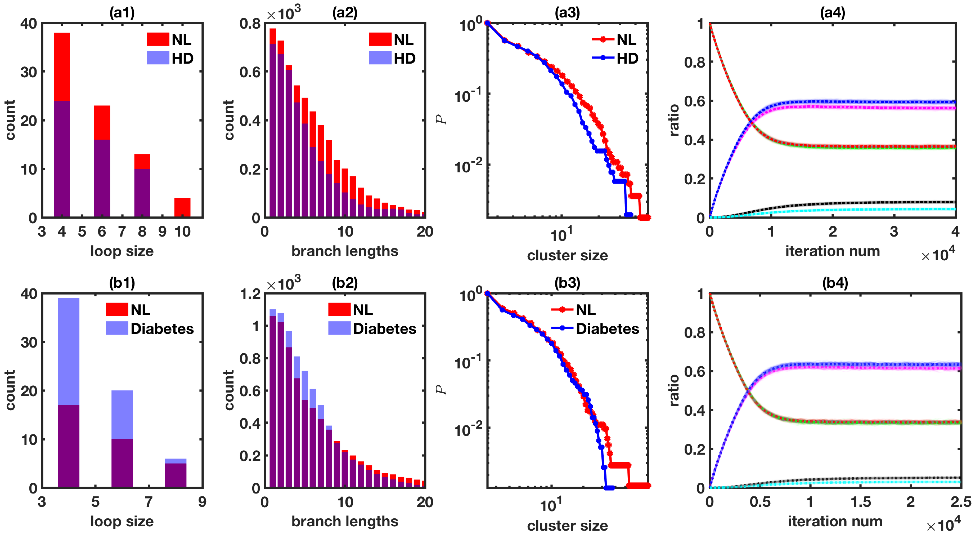

Supplement: S2 Fig — Here we compare mitochondrial network fragmentation in HD (striatal cells from mouse embryos bearing a 111 polyglutamine repeat Q111/0 and Q111/1) versus control [78] with C2n/C2d = 2.0 (top row) and diabetes (MitoNEET knockout mouse embryonic fibroblasts) versus control [65] with C2n/C2d = 1.8 (bottom row). Distributions of (a1) loop sizes, (a2) branch lengths, and (a3) cluster sizes (cumulative probability) for NL (red) and diseased cells (blue) from experimental images. (a4) Fraction of X1 (NL: green, diseased: red), X2 (NL: magenta, diseased: blue) and X3 (NL: black, diseased: cyan) species from the model as functions of the number of iterations using C1 and C2 values for HD experiments. The model results show average of 100 runs. (b1-b4) shows the same mitochondrial network features as (a1-a4) for MitoNEET knockout mouse embryonic fibroblasts with diabetes pathology and their normal counterparts. (TIFF) [file pone.0223014.s003.tiff]

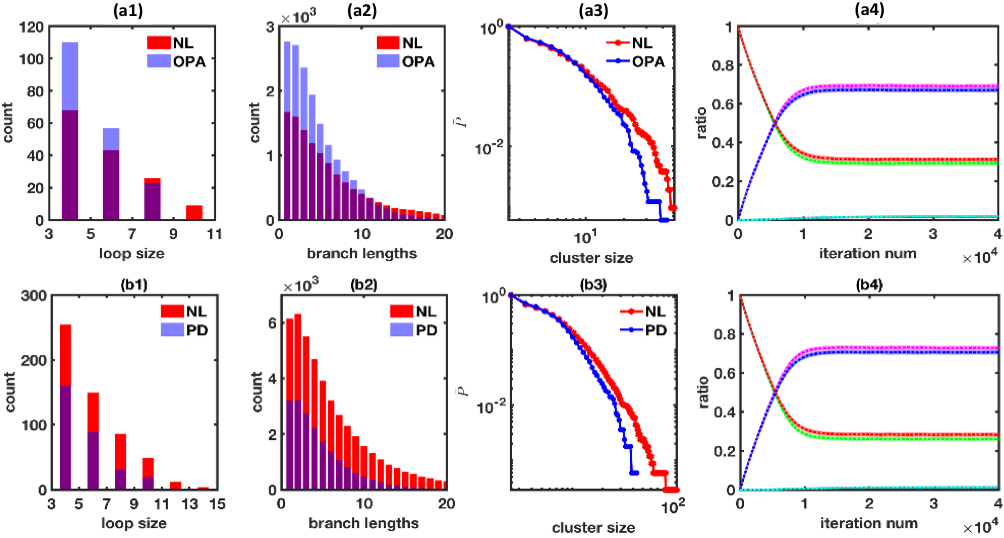

Supplement: S3 Fig — Here we compare mitochondrial network fragmentation in OPA (mouse embryonic fibroblasts with the pathogenic mutation R149W in human YME1L1) versus control [66] with C1n/C1d = 1.18 (top row) and PD (human primary skin fibroblasts obtained from sporadic late-onset PD patients) versus those from healthy age-matched control subjects [81] with C1n/C1d = 1.22 (bottom row). Distributions of (a1) loop sizes, (a2) branch lengths, and (a3) cluster sizes (cumulative probability) for NL (red) and diseased cells (blue) from experimental images. (a4) Fraction of X1 (NL: green, diseased: red), X2 (NL: magenta, diseased: blue) and X3 (NL: black, diseased: cyan) species from the model as functions of the number of iterations using C1 and C2 values for OPA experiments. The model results show average of 100 runs. (b1-b4) shows the same mitochondrial network features as (a1-a4) for human primary skin fibroblasts with PD pathology and their normal counterparts. Note that the curves for X3 species in diseased and normal cells overlap (a4, b4). (TIFF) [file pone.0223014.s004.tiff]
