# Supplementary material for: Mitochondrial fragmentation and network architecture in degenerative diseases
Source: PLoS One. 2019 Sep 26;14(9):e0223014. doi: 10.1371/journal.pone.0223014 (PMC6762132; doi:10.1371/journal.pone.0223014)
Supplement: S1 Table — Column 1 provides the disease, column 3 reports the cell/animal model, column 4 lists the condition for the experiment (normal versus diseased), and column 5 provides references where the images were originally published. Abbreviations: WT-Wild type, KO-Knockout, NL-Normal, MEF-Mouse embryonic fibroblasts, HSF-Human skin fibroblasts, MEMN Mouse embryonic motor neurons, TM-YAC128 Transgenic mice Yeast Artificial Chromosome 128, HSF Human Skin Fibroblasts, RPTCs rat proximal tubular cells, RCA rat cortical astrocytes, CHCHD10—Coiled-coil-helix-coiled-coil-helix domain-containing protein 10, YME1L1—Yeast mitochondrial escape 1-like 1 gene. (DOCX) [file pone.0223014.s005.docx]

**S1 Table**

| Condition | | Normal vs Diseased | Cell/Animal Model | Experiment | Reference |
| --- | --- | --- | --- | --- | --- |
| HD | | NL | TM-YAC128 | control | [1] |
|  |  | HD |  | Polyglutamate 111 repeat |  |
| AD | 1 | NL | M17 | APP WT | [2] |
|  |  | AD |  | APPswe (swedish mutant) |  |
|  | 2 | NL | HSF | Healthy | [3] |
|  |  | AD |  | AD-HSF |  |
| DS | | NL | HF | NL-HF | [4, 5] |
|  |  | DS |  | DS-HF |  |
| PD | 1 | NL | HSF | NL-HSF | [6] |
|  |  | PD |  | PD-HSF |  |
|  | 2 | NL | MEF | DJ-1 WT | [7] |
|  |  | PD |  | DJ-1 KO |  |
| ALS | 1 | NL | C.elegens | CHCHD10 WT | [8] |
|  |  | ALS |  | R15L mutation |  |
|  | 2 | NL | MEMN E13.5 | TDP-43 WT | [9] |
|  |  | ALS |  | ALS mutant TDP-43 |  |
| Kidney injury | | NL | RPTCs | NL | [10] |
|  |  | Kidney injury |  | Azide |  |
| Diabetes/Cancer | | NL | MEF | NL | [11] |
|  |  | Diabetes/Cancer |  | MitoNEET KO |  |
| OPA | | NL | MEF | YME1L1 WT | [12] |
|  |  | OPA |  | YME1L1 KO |  |
| Ca2+ | | NL | RCA | RCA NL | [13] |
|  |  | Ca^2+^ overload |  | RCA 4Br-A23187 treated |  |

1. Costa V, Giacomello M, Hudec R, Lopreiato R, Ermak G, Lim D, et al. Mitochondrial fission and cristae disruption increase the response of cell models of Huntington's disease to apoptotic stimuli. EMBO molecular medicine. 2010;2(12):490-503.

2. Wang X, Su B, Siedlak SL, Moreira PI, Fujioka H, Wang Y, et al. Amyloid-β overproduction causes abnormal mitochondrial dynamics via differential modulation of mitochondrial fission/fusion proteins. Proceedings of the National Academy of Sciences. 2008;105(49):19318-23.

3. Perez MJ, Ponce DP, Osorio-Fuentealba C, Behrens MI, Quintanilla RA. Mitochondrial Bioenergetics Is Altered in Fibroblasts from Patients with Sporadic Alzheimer's Disease. Front Neurosci-Switz. 2017;11. doi: ARTN 553

10.3389/fnins.2017.00553. PubMed PMID: WOS:000412323100001.

4. Zamponi E, Zamponi N, Coskun P, Quassollo G, Lorenzo A, Cannas SA, et al. Nrf2 stabilization prevents critical oxidative damage in Down syndrome cells. Aging Cell. 2018;17(5). doi: UNSP e12812

10.1111/acel.12812. PubMed PMID: WOS:000445599100008.

5. Izzo A, Nitti M, Mollo N, Paladino S, Procaccini C, Faicchia D, et al. Metformin restores the mitochondrial network and reverses mitochondrial dysfunction in Down syndrome cells. Human molecular genetics. 2017;26(6):1056-69.

6. Teves JM, Bhargava V, Kirwan KR, Corenblum MJ, Justiniano R, Wondrak GT, et al. Parkinson's Disease Skin Fibroblasts Display Signature Alterations in Growth, Redox Homeostasis, Mitochondrial Function, and Autophagy. Frontiers in neuroscience. 2018;11:737.

7. Krebiehl G, Ruckerbauer S, Burbulla LF, Kieper N, Maurer B, Waak J, et al. Reduced Basal Autophagy and Impaired Mitochondrial Dynamics Due to Loss of Parkinson's Disease-Associated Protein DJ-1. Plos One. 2010;5(2). doi: ARTN e9367

10.1371/journal.pone.0009367. PubMed PMID: WOS:000274924000010.

8. Woo J-A, Liu T, Trotter C, Fang CC, De Narvaez E, LePochat P, et al. Loss of function CHCHD10 mutations in cytoplasmic TDP-43 accumulation and synaptic integrity. Nature Communications. 2017;8:15558.

9. Wang W, Li L, Lin W-L, Dickson DW, Petrucelli L, Zhang T, et al. The ALS disease-associated mutant TDP-43 impairs mitochondrial dynamics and function in motor neurons. Human molecular genetics. 2013;22(23):4706-19.

10. Brooks C, Wei Q, Cho S-G, Dong Z. Regulation of mitochondrial dynamics in acute kidney injury in cell culture and rodent models. The Journal of clinical investigation. 2009;119(5):1275-85.

11. Molina AJ, Wikstrom JD, Stiles L, Las G, Mohamed H, Elorza A, et al. Mitochondrial networking protects beta cells from nutrient induced apoptosis. Diabetes. 2009.

12. Hartmann B, Wai T, Hu H, MacVicar T, Musante L, Fischer-Zirnsak B, et al. Homozygous YME1L1 mutation causes mitochondriopathy with optic atrophy and mitochondrial network fragmentation. Elife. 2016;5:e16078.

13. Deheshi S, Dabiri B, Fan S, Tsang M, Rintoul GL. Changes in mitochondrial morphology induced by calcium or rotenone in primary astrocytes occur predominantly through ros-mediated remodeling. Journal of Neurochemistry. 2015;133(5):684-99. doi: 10.1111/jnc.13090. PubMed PMID: WOS:000353570500007.
