# Supplementary material for: Mitochondrial fragmentation and network architecture in degenerative diseases
Source: PLoS One. 2019 Sep 26;14(9):e0223014. doi: 10.1371/journal.pone.0223014 (PMC6762132; doi:10.1371/journal.pone.0223014)
Supplement: S2 Table — Column 1 lists the condition for which images of normal (NL) and diseased cells were analyzed (column 2). Columns 3 & 4 and 5 & 6 compare and Ng/N respectively from experiment and theory. Columns 7 & 8 are the C1 (tip-to-tip fusion/fission) and C2 (tip-to-side fusion/fission) values obtained by fitting the model to the data and used in simulations. (DOCX) [file pone.0223014.s006.docx]

**S2 Table**

| Condition | | Normal vs diseased | Mean degree <k>  Exp Theory | | N_g_/N  Exp Theory | | C_1_ | C_2_ |
| --- | --- | --- | --- | --- | --- | --- | --- | --- |
| AD | 1 | NL | 1.64 | 1.64 | 0.042 | 0.108 | 7.0e-4 | 2.30e-4 |
|  |  | AD | 1.62 | 1.62 | 0.038 | 0.067 | 7.0e-4 | 1.90e-4 |
|  | 2 | NL | 1.67 | 1.67 | 0.012 | 0.050 | 4.0e-4 | 6.50e-5 |
|  |  | AD | 1.65 | 1.65 | 0.016 | 0.025 | 4.0e-4 | 5.00e-5 |
| PD | 1 | NL | 1.72 | 1.72 | 0.006 | 0.008 | 1.2e-3 | 7.00e-6 |
|  |  | PD | 1.70 | 1.70 | 0.005 | 0.007 | 9.8e-4 | 7.00e-6 |
|  | 2 | NL | 1.47 | 1.47 | 0.0074 | 0.029 | 7.0e-5 | 9.00e-5 |
|  |  | PD | 1.45 | 1.45 | 0.0048 | 0.013 | 7.0e-5 | 8.00e-5 |
| ALS | 1 | NL | 1.75 | 1.75 | 0.369 | 0.359 | 4.8e-4 | 1.00e-4 |
|  |  | ALS | 1.69 | 1.69 | 0.222 | 0.225 | 1.0e-4 | 1.00e-4 |
|  | 2 | NL | 1.69 | 1.69 | 0.030 | 0.034 | 9.0e-4 | 7.00e-5 |
|  |  | ALS | 1.67 | 1.67 | 0.041 | 0.032 | 8.0e-4 | 7.00e-5 |
